# An RNA–RNA crosstalk network involving HMGB1 and RICTOR facilitates hepatocellular carcinoma tumorigenesis by promoting glutamine metabolism and impedes immunotherapy by PD-L1+ exosomes activity

**DOI:** 10.1038/s41392-021-00801-2

**Published:** 2021-12-17

**Authors:** Yanping Wei, Xuewu Tang, Yibin Ren, Yun Yang, Fengliang Song, Jingbo Fu, Shuowu Liu, Miao Yu, Jing Chen, Suyang Wang, Kecheng Zhang, Yexiong Tan, Zhipeng Han, Lixin Wei, Baohua Zhang, Zhangjun Cheng, Liang Li, Hongyang Wang

**Affiliations:** 1 International Co-operation Laboratory on Signal Transduction, Eastern Hepato-Biliary Surgery Institute, Second Military Medical University, Shanghai, China; 2 National Center for Liver Cancer, Shanghai, China; 3 Hepato-Pancreato-biliary center, Zhongda Hospital, School of Medicine, Southeast University, Nanjing, China; 4 The Third Department of Hepatic Surgery, Eastern Hepatobiliary Surgery Hospital, Shanghai, China; 5 School of Medicine, Nantong University, Nantong, Jiangsu Province China; 6 Department of Biliary Tract Surgery, Eastern Hepatobiliary Surgery Hospital, Second Military Medical University, Shanghai, China; 7 Tumor Immunology and Gene Therapy Center, Eastern Hepato-Biliary Surgery Institute, Second Military Medical University, Shanghai, China; 8 National Laboratory for Oncogenes and Related Genes, Cancer Institute, RenJi Hospital, Shanghai Jiao Tong University, 200441 Shanghai, China

**Keywords:** Cancer, Gastrointestinal cancer

## Abstract

Hepatocellular carcinoma (HCC) is the global leading cause of cancer-related deaths due to the deficiency of targets for precision therapy. A new modality of epigenetic regulation has emerged involving RNA–RNA crosstalk networks where two or more competing endogenous RNAs (ceRNAs) bind to the same microRNAs. However, the contribution of such mechanisms in HCC has not been well studied. Herein, potential HMGB1-driven RNA–RNA crosstalk networks were evaluated at different HCC stages, identifying the mTORC2 component RICTOR as a potential HMGB1 ceRNA in HBV^+^ early stage HCC. Indeed, elevated HMGB1 mRNA was found to promote the expression of RICTOR mRNA through competitively binding with the miR-200 family, especially miR-429. Functional assays employing overexpression or interference strategies demonstrated that the HMGB1 and RICTOR 3′untranslated regions (UTR) epigenetically promoted the malignant proliferation, self-renewal, and tumorigenesis in HCC cells. Intriguingly, interference against HMGB1 and RICTOR in HCC cells promoted a stronger anti-PD-L1 immunotherapy response, which appeared to associate with the production of PD-L1^+^ exosomes. Mechanistically, the HMGB1-driven RNA-RNA crosstalk network facilitated HCC cell glutamine metabolism via dual mechanisms, activating a positive feedback loop involving mTORC2-AKT-C-MYC to upregulate glutamine synthetase (GS) expression, and inducing mTORC1 signaling to derepress SIRT4 on glutamate dehydrogenase (GDH). Meanwhile, this crosstalk network could impede the efficacy of immunotherapy through mTORC1-P70S6K dependent PD-L1 production and PD-L1^+^ exosomes activity. In conclusion, our study highlights the non-coding regulatory role of HMGB1 with implications for RNA-based therapeutic targeting together with a prediction of anti-PD-L1 immunotherapy in HCC.

## Introduction

Hepatocellular carcinoma (HCC) accounts for nearly 90% of primary liver cancers and represents a leading cause of cancer-related deaths worldwide^1^. It has been estimated that if HCCs could be identified in the early stages, treatment by surgical resection would result in 5-year survival rates exceeding 70%^2^. However, most HCC patients are diagnosed late due to insufficient surveillance. Without effective treatment options for advanced HCC, survival outcomes are dire, for example, 5-year survival rates in China are less than 12.5%^2,3^. Hence, understanding the biological mechanisms involved in HCC tumorigenesis and the molecular phenotype of early stage HCC is mandatory for improving early diagnosis and developing curative treatments.

MicroRNAs negatively regulate the stability and translation of target RNAs through the RNA-induced silencing complex (RISC)^4^. Emerging evidence supports the competitive endogenous RNA (ceRNA) hypothesis that different RNAs harboring the same microRNA response elements (MREs) compete for the same pool of microRNAs. Consequently, the competition amongst different target RNAs for microRNA binding can titrate the microRNAs and de-repress their target transcripts^5^. Notably, this allows the RNA transcripts of protein-coding genes to function independently of their protein translation potential as non-coding genes to regulate other genes^6^. The growing experimental evidence for this “RNA–RNA” crosstalk mechanism has shed new light on novel coding-independent interactions and challenges the traditional “protein–protein”-dominated view of gene regulation.

CeRNA activity plays an important role in physiological and pathological processes including growth, development, and cancer. As an example, the important tumor suppressor gene PTEN is contained within a huge ceRNA network^7^. Among other interactions, PTEN RNA interacts with its pseudogene PTENP1 through common microRNAs and when such crosstalk goes work, the ceRNA network leads to inhibition of PTEN expression and activation of the PI3K/Akt pathway to promote tumor progression. In addition, the CD44 3′untranslated region (3′UTR) regulates the expression of CD44 protein and the cell cycle regulator CDC42 by competing with miR-216, miR-330, and miR-608^8^. Pseudogene KRAS1P is considered to be the ceRNA of the proto-oncogene KRAS, which can enrich KRAS transcripts to promote cell growth^5^. Along with coding gene transcripts and pseudogenes, noncoding RNAs can also interact with other genes as ceRNAs. For instance, the long noncoding RNA LINC-MD1 regulates the expression of MAML1 and MEF2C proteins by competitively binding miR-133 and miR-135 to affect muscle development^9^. Circular RNA Circ-CDYL was found to interact with mRNAs encoding HDGF and HIF1AN by acting as the sponge of miR-892a and miR-328-3p, respectively, thus promoting the early occurrence of HCC^10^.

The high-mobility group box 1 gene (HMGB1), located on chromosome 1q32, encodes a very abundant non-histone type nuclear protein, which is expressed in almost all the cells and tissues. It was previously thought to act as a nuclear factor that enhances transcription as was considered the “nuclear weapon” in the immunity arsenal. More recently, HMGB1 was discovered to be a crucial cytokine that mediates pathological effects in sepsis, arthritis, cancer, and other diseases^11^. Our previous studies revealed HMGB1-driven signaling mechanisms in several types of liver diseases. We found that TLR4/MyD88 signaling in liver parenchymal cells plays a pivotal role during the early progression of HFD-induced nonalcoholic fatty liver disease (NAFLD), in which free HMGB1 served as a positive component mediating TLR4 activation^12^. Interestingly, HMGB1 is regulated by both JNK1/2-ATF2-dependent signaling at the transcriptional level, and by the miR-200 family, at the post-transcriptional level in nonalcoholic steatohepatitis (NASH)^13^. Moreover, we found that hepatitis B virus X protein can induce the translocation and secretion of HMGB1 to promote the malignant progression of HCC in an autocrine/paracrine manner, and HMGB1 released by tumor cells plays a critical role in TLR4-dependent activation of platelets which in turn promotes HCC metastasis^14,15^. These studies propose HMGB1 as a promising biomarker and potential therapeutic target of NAFLD and HBV-related HCC, but presently the epigenetic functions associated with HMGB1 in HCC remain unknown.

Here, we studied how HMGB1, independent of protein-coding function, exerts regulatory effects in HBV^+^ early stage HCC through an RNA-RNA crosstalk mechanism involving the miR-200 family targeting sites. Furthermore, we explored the potential mechanism of HMGB1 as a ceRNA crosstalk with RICTOR facilitates the stemness characteristics and tumorigenesis of HCC by promoting glutamine metabolism. Moreover, this mechanism can impede the efficacy of immunotherapy through PD-L1 production and PD-L1^+^exosomes activity.

## Results

### RICTOR is a key putative ceRNA in the HMGB1-related RNA crosstalk network in HBV^+^ early stage HCC

**Fig. 1:**
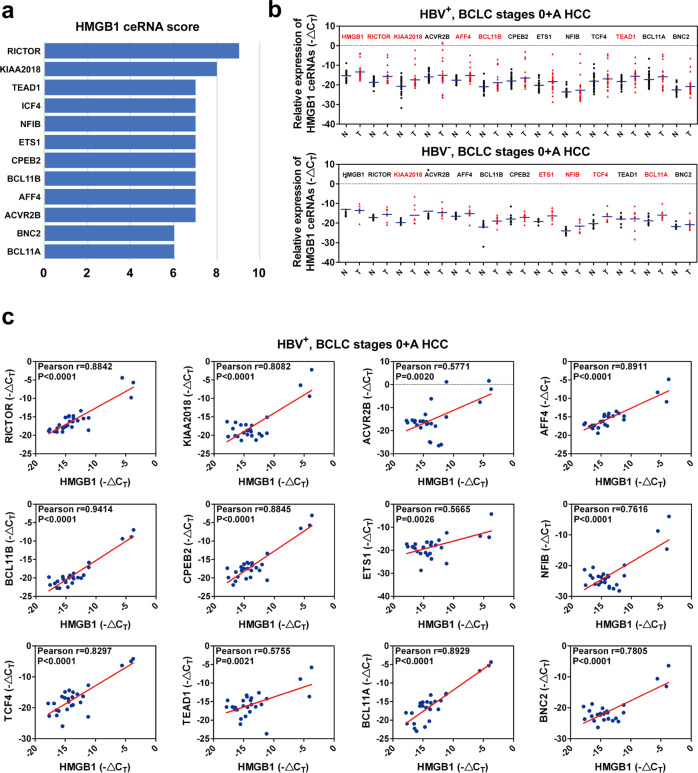
HMGB1 epigenetically crosstalks with RICTOR in early stage HCC. **a** Prediction of candidate HMGB1 crosstalk RNAs using the ceRDB database (https://www.oncomir.umn.edu/cefinder). The top 12 candidate RNAs and scores were shown. **b** The RNA expression levels of HMGB1 and the 12 candidate RNAs were analyzed in early stage HCC (BCLC stages 0 and A HCC) tissues by qRT-PCR. HBV^+^ HCC (above) *n* = 26; HBV^−^ HCC (below), *n* = 9. The results are means ± SD. Names marked red and black represent significantly (*p* < 0.05) and nonsignificant genes, respectively. **c** Pearson correlation coefficient analysis between expression levels of HMGB1 and the 12 candidate RNAs, respectively, in early stage HCC tumor tissues

To identify the potential target transcripts associated with HMGB1 in an RNA–RNA crosstalk network, we first perform bioinformatics analysis using the ceRDB database (https://www.oncomir.umn.edu/cefinder). Notably, of the 12 candidate ceRNAs of HMGB1 identified, RICTOR provided the highest scoring match (Fig. 1a).

The primary evidence shows that RNA transcripts co-exist within a ceRNA network involves their coregulation in relevant biological samples^5^. To determine the expression relationships between HMGB1 and the candidate ceRNAs, we next used qRT-PCR to measure transcript levels in HCC clinical samples. The results of these assays showed that HMGB1 was co-upregulated with RICTOR, KIAA2018, AFF4, BCL11B, and TEAD1 in HBV^+^ early-stage HCC (BCLC stages 0 + A HCC) (Fig. 1b, top). In contrast, in HBV^−^ early stage HCC tissues, there was no tendency for HMGB1 and RICTOR to be highly expressed in tumors (Fig. 1b, bottom). Further, Pearson correlation coefficients analysis determined that HMGB1 expression was significantly correlated with RICTOR, KIAA2018, AFF4, BCL11B, and TEAD1 levels in HBV^+^ early stage HCC (Fig.1c). These results proposed that HMGB1 was involved in an RNA–RNA regulatory network in HBV^+^ HCC with expression evidence showing likely crosstalk with five different coding genes.

The mTORC2-AKT signal transduction pathway plays an important role in the occurrence and development of liver cancer. Activation of mTORC2 phosphorylates AKT at Ser473, thereby activating a series of downstream signaling pathways^16^. Since RICTOR provided the highest scoring match and acts as an important component of the mTORC2, we prioritized RICTOR for our subsequent investigations into the ceRNA regulation of HMGB1. Instructively, further analysis of HMGB1 and RICTOR mRNA levels in HBV^+^ advanced stage HCC (BCLC stages B + C HCC) found both transcripts were upregulated. However, unlike in HBV^+^ early stage HCC, there was no significant correlation between their expression levels in the advanced cases (Supplementary Fig. 1a, b). These results indicated that the proposed ceRNA regulation between HMGB1 and RICTOR mRNAs might be specific in HBV^+^ early stage HCC.

### HMGB1 regulates the expression of RICTOR in HCC cells via ceRNA crosstalk

**Fig. 2:**
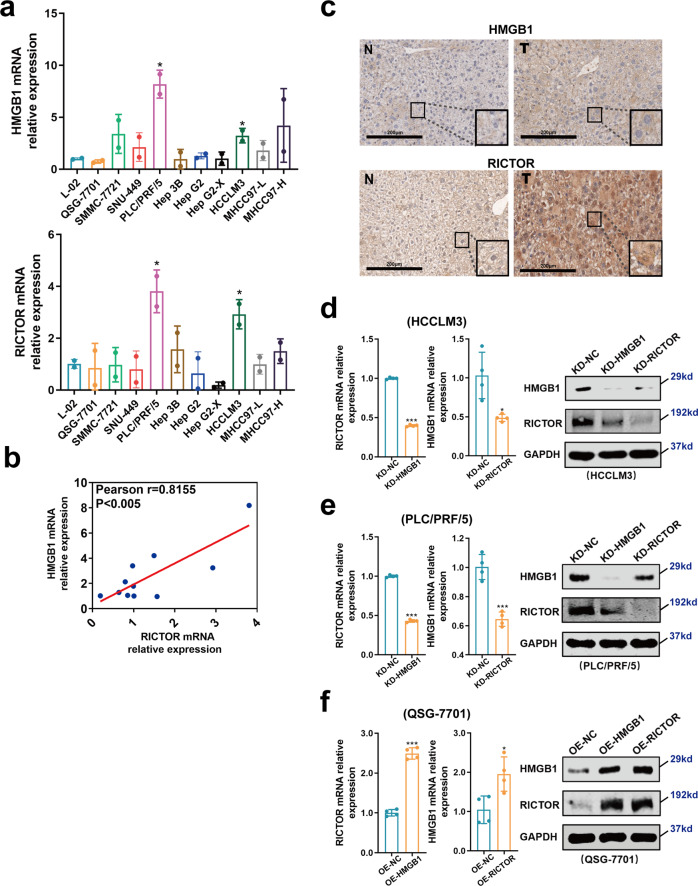
HMGB1 epigenetically regulates the expression level of RICTOR in HCC. **a** The mRNA expression of HMGB1 and RICTOR in the 11 indicated cell lines as determined by qRT-PCR. *p < 0.05. **b** Pearson correlation coefficient analysis between the expression level of HMGB1 and RICTOR in the cell lines from (**a**). **c** Immunohistochemical staining of HMGB1 and RICTOR in liver tissues in the DEN + CCl4-induced liver cancer mouse model. **d**, **e** The expression levels of RICTOR and HMGB1 mRNA (left) and protein (right) after the interference of HMGB1 or RICTOR in the HCCLM3 (**d**) and PLC/PRF/5 (**e**) cell lines (abbreviated as “KD-HMGB1 or KD-RICTOR”, respectively). **f** The expression levels of RICTOR and HMGB1 mRNA (left) and protein (right) after overexpression of HMGB1 or RICTOR 3′UTR in the QSG-7701 cell line (abbreviated as “OE-HMGB1 or OE-RICTOR”, respectively). The mRNA levels (left) were determined by qRT-PCR and protein level (right) was determined by Western blot. *p < 0.05, ***p < 0.001

We next undertook analysis in HCC cell lines to better delineate if HMGB1 RNA exerts protein-coding-independent regulation of RICTOR. We first compared the mRNA expression levels of HMGB1 and RICTOR in human HCC cell lines versus immortalized normal liver cells. We found that both HMGB1 and RICTOR were highly expressed in HCCLM3 and PLC/PRF/5 HCC cell lines, but with relatively lower expression in L-02 and QSG-7701 normal liver cells (Fig. 2a). Moreover, Pearson correlation coefficient analysis showed that the expression levels of HMGB1 mRNA were positively correlated with RICTOR mRNA in a panel of 11 liver-derived cell lines (Fig. 2b). To further examine the expression levels of HMGB1 and RICTOR in the experimental context, we utilized the DEN + CCl4-induced liver cancer model in C57 mice. Immunohistochemical analysis showed that HMGB1 and RICTOR were coupregulated in HCC tissues compared to matched normal adjacent tissues (Fig. 2c).

Based on the relative expression levels observed in the different normal and HCC cell lines, we used lentiviral transduction to construct stable HMGB1 and RICTOR 3′UTR over-expressing cell lines in normal QSG-7701 liver cells (OE-HMGB1/RICTOR cells), whereas specific shRNA was used to stably knockdown HMGB1/RICTOR in HCCLM3 cell line, respectively, or in PLC/PRF/5 HCC cell line (KD-HMGB1/RICTOR cells) (Supplementary Fig. 2a). Characterization of the manipulated cell lines using qRT-PCR and Western blotting showed that HMGB1 interference and overexpression of HMGB1 3′UTR respectively reduced and increased the expression level of RICTOR at both RNA and protein levels (Fig. 2d–f, Supplementary Fig. 2b). Similarly, RICTOR interference and 3′UTR overexpression resulted in the downregulation and upregulation of HMGB1, respectively (Fig. 2d–f, Supplementary Fig. 2b).

Taken together, these results indicated that HMGB1 regulates the expression of RICTOR in liver cells and HCC cells in a ceRNA crosstalk manner.

### HMGB1 regulates RICTOR expression in HCC by competitively binding to the miR-200 family

“RNA–RNA” crosstalk relies on mRNAs functioning as “microRNA sponges”, which can relieve the inhibitory effect of the microRNAs on their target ceRNAs^7^. HMGB1 3′UTR harbors conserved MREs for the miR-200 family, which has previously been elucidated to constrain the NASH^13^. CLIP database (http://starbase.sysu.edu.cn/) revealed that RICTOR along with HMGB1 may be targeted by the miR-200 family (supplementary Fig. 2c). In addition, when downregulated miR-200 family members with antagomir, the “RNA–RNA” crosstalk between HMGB1 and RICTOR were disturbed (Supplementary Fig. 2d).

**Fig. 3:**
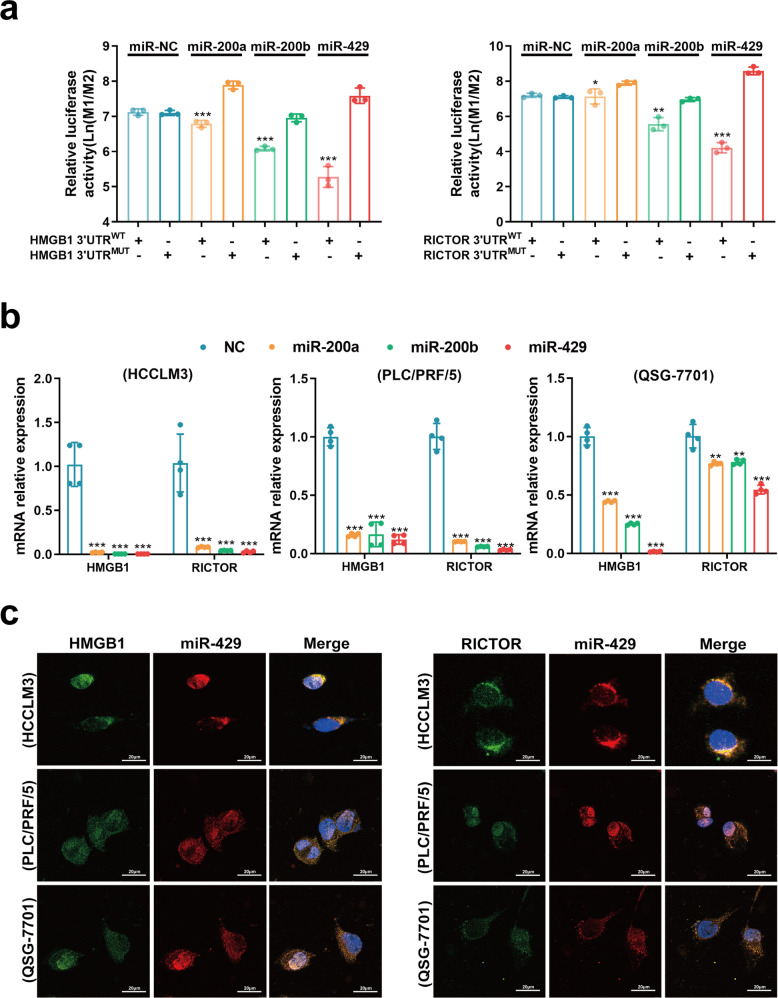
miR-200 family mediates the “RNA–RNA” crosstalk between HMGB1 and RICTOR. **a** Luciferase assays with reporter constructs containing the wild-type or mutant HMGB1/RICTOR 3′UTR downstream of the luciferase gene were performed after co-transfection with miR-200a/200b/429 mimics in HEK293T cells. *p < 0.05, **p < 0.01, ***p < 0.001. **b** The mRNA levels of HMGB1 and RICTOR were determined by qRT-PCR 48 h after transfection of 100 nM miR-200a/200b/429 mimics in indicated cells. **p < 0.01, ***p < 0.001. **c** RNA FISH assays detecting the cellular localization of HMGB1/RICTOR and miR-429 in indicated cells

To verify whether HMGB1 and RICTOR are both target genes of the miR-200 family (miR-200a/200b/429) in HCC, luciferase reporter gene plasmids were constructed with either wild-type 3′UTR (3′UTR^WT^) or mutated binding sites of miR-200a/200b/429 in 3′UTR (3′UTR^MUT^) of HMGB1 and RICTOR mRNAs. Notably, after HEK-293T cells were transfected with the reporter gene plasmids in combination with miR-200a/200b/429 mimics, respectively, we found that miR-200a/200b/429 inhibited the activity of both HMGB1 and RICTOR 3′UTR^WT^ constructs but not the 3′UTR^MUT^ equivalents (Fig. 3a). Moreover, consistent with the ability of HMGB1 and RICTOR mRNAs to be targeted by the miR-200 family, transfection of miR-200a/200b/429 mimics remarkably inhibited their mRNA expression levels (Fig. 3b). Intriguingly, among the miR-200 family, miR-429 had the most significant inhibitory effect on HMGB1 and RICTOR at mRNA levels (Fig. 3a, b).

Lastly, to provide independent evidence for the ceRNA mechanism, we used RNA fluorescence in situ hybridization (FISH) to determine the localization of HMGB1, RICTOR, and miR-429 in HCC cells. Co-localization between HMGB1, RICTOR, and miR-429 in HCCLM3, PLC/PRF/5, and QSG-7701 cells (Fig. 3c), clearly provided evidence for binding between miR-429, HMGB1, and RICTOR in situ.

In all, our data indicated that up-regulated HMGB1 mRNA expression in HCC functions as a microRNA sponge to competitively bind the miR-200 family, thereby increasing the expression of RICTOR mRNA.

### HMGB1 and RICTOR mRNAs epigenetically promote stemness characteristics and tumorigenesis in HCC

**Fig. 4:**
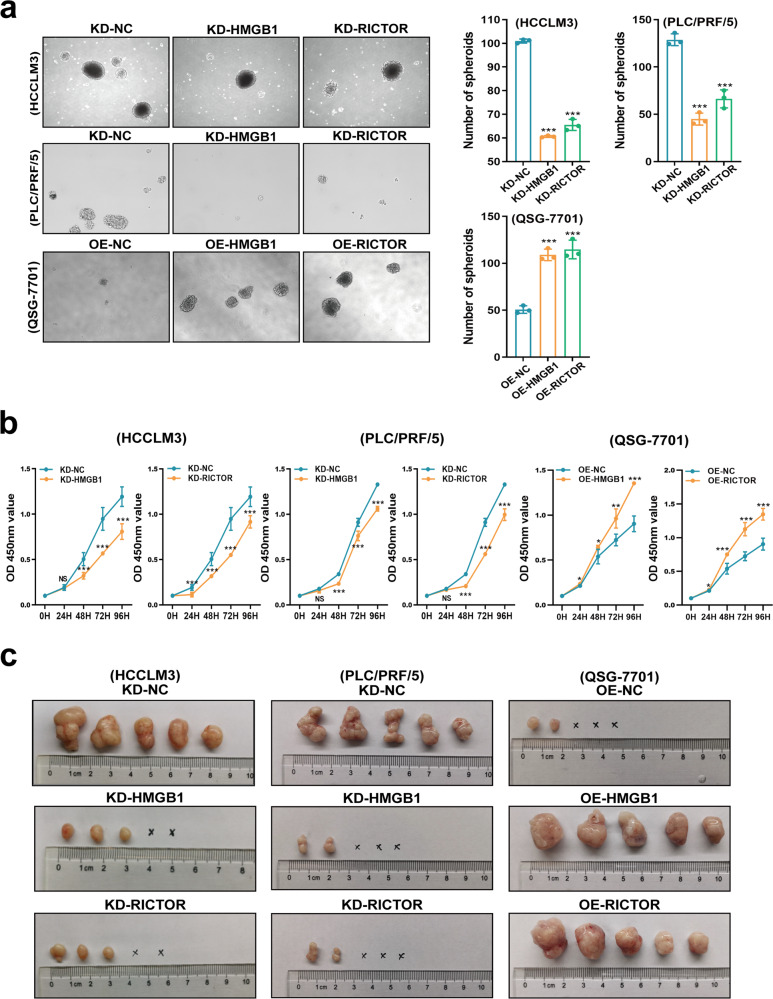
HMGB1 epigenetically promotes stem-like properties in HCC cells. **a** Determination of spheroid formation after 3000 cells were seeded in low-adhesion plates for 10 days (left). The number of tumor spheroids was quantitated (right). ***p < 0.001. **b** Cell proliferation was evaluated using CCK8 assays. The results are means ± SEM. *p < 0.05, **p < 0.01, ***p < 0.001. **c** Male nude mice (*n* = 5) were subcutaneously injected with 1 × 10^4^ HMGB1/RICTOR 3′UTR over-expressed liver cells (abbreviated as “OE-HMGB1 or OE-RICTOR”, respectively) or HMGB1/RICTOR mRNA interference HCC cells (abbreviated as “KD-HMGB1 or KD-RICTOR”, respectively). Tumorigenesis was assessed after 48 days as shown

In view of the high expression of HMGB1 mRNA detected in early stage HCC, we further explored the contribution of HMGB1 RNA to HCC stemness characteristics and tumorigenesis. Using the HMGB1 and RICTOR-manipulated cell lines we found that downregulation of HMGB1 and RICTOR mRNAs significantly inhibited tumor spheroid growth and cell proliferation in vitro (Fig. 4a, b). Spheroids were collected to evaluate the cancer stem cells (CSCs) markers, such as EPCAM, CD133, and CD24, in HMGB1/RICTOR manipulated cells using real-time PCR methods. Overexpression of HMGB1/RICTOR 3′UTR in liver cells (OE-HMGB1/RICTOR groups) significantly increased the levels of CSCs markers (supplementary Fig. 3a). Conversely, HMGB1/RICTOR interference HCC cells (KD-HMGB1/RICTOR groups) showed lower levels of CSC markers compared to the negative controls (Supplementary Fig. 3a). Moreover, in vivo growth was similarly inhibited in subcutaneously implanted tumors (Fig. 4c and Supplementary Fig. 3b, c) with decreased proportions of EPCAM^+^ liver tumor-stem cells detected (Supplementary Fig. 3d). Conversely, ectopic overexpression of HMGB1 and RICTOR 3′UTR led to growth and stemness-promoting phenotypes in both the in vitro and in vivo models (Fig. 4 and Supplementary Fig. 3a–d).

In addition, to further identify the mTORC2 manipulating effects in HMGB1 interference HCC cells (KD-HMGB1 group), we transfected RICTOR-overexpressed plasmid into the HMGB1 stably interfered HCCLM3 and PLC/PRF/5 cell lines, respectively, and confirmed the mechanism by repeating key experiments using these HCC cells. Overexpression of RICTOR rescued the impeded malignant proliferation of KD-HMGB1 HCC cells (Supplementary Fig. 3e). These results indicated that HMGB1 mRNA epigenetically promoted tumor growth via an mTORC2-dependent pathway.

To more solidly identify what HMGB1 and RICTOR “RNA–RNA” crosstalk does in HCC cells, we transfected QSG-7701 cell line with HMGB1/RICTOR 3′UTR-overexpression plasmid harboring muted binding sites of miR-200 family. The cells transfected with sites-muted HMGB1/RICTOR 3′UTR plasmids (OE-HMGB1/RICTOR 3′UTR^MUT^ groups) presented similar stemness property and proliferation to the negative control cells (OE-NC group, Supplementary Fig. 3f, g), respectively. These results indicated that HMGB1 and RICTOR mRNAs epigenetically promote tumorigenesis in HCC, which depends on the miR-200 family binding sites in 3′UTR.

Taken together, these results established that HMGB1 and RICTOR mRNAs can promote HCC stemness characteristics and contribute to tumorigenesis via RNA–RNA crosstalk.

### HMGB1 and RICTOR mRNAs epigenetically impede the response to anti-PD-L1 immunotherapy via upregulation of PD-L1^+^ exosomes in HCC

**Fig. 5:**
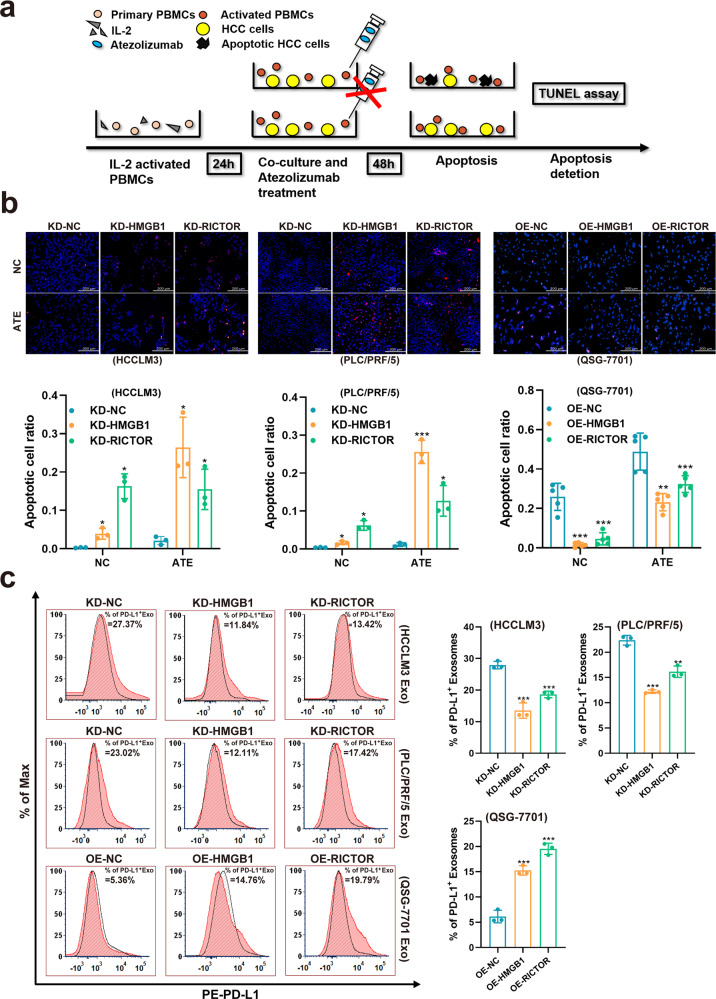
HMGB1 and RICTOR mRNAs epigenetically impede responses to anti-PD-L1 therapy in HCC through PD-L1^+^exosomes activity. **a** PMBC killing assay scheme. HMGB1/RICTOR mRNA interference HCC cells (abbreviated as “KD-HMGB1 or KD-RICTOR”, respectively) were cocultured with activated PBMCs and treated with or without Atezolizumab for 48 h before apoptosis detection. **b** Cell apoptosis in treated HCC cells was evaluated by TUNEL assay (above). The apoptotic cell ratios were shown (below). **c** Exosomes isolated from cell culture supernatants in treated HCC cells were determined by flow cytometry using PE-fluorescein-conjugated anti-PD-L1 antibody (red). Isotype matched antibody (PE-fluorescein-conjugated anti-IgG antibody) was used as gating controls (black). Percentages of PD-L1^+^ Exo resulted from the relative fluorescence values gated by isotype controls. Exo: exosomes. **p < 0.01, ***p < 0.001

Therapeutic resistance is one of the characteristics of tumor stemness, so we next explored whether the “RNA–RNA” crosstalk of HMGB1 and RICTOR can influence the therapeutic response of HCC. The elevated expression of inhibitory immune checkpoints is related to T cell-mediated immune responses in cancer immunotherapy^17^. Similar to previous clinical findings^18^, we observed aberrantly high expression levels of PD-L1 in both early and late-stage HCC (Supplementary Fig. 4a). This observation established the rationale for the administration of anti-PD-L1 immunotherapy in early stage HCC. In particular, we examined how HMGB1 mRNA expression affected T-cell cytotoxicity against HCC in the presence of anti-PD-L1 treatment. Indeed, the addition of atezolizumab (anti-PD-L1 antibody) to a peripheral blood mononuclear cells (PBMCs) coculture system with HMGB1/RICTOR interference HCC cell lines (Fig. 5a) showed comparatively increased numbers of apoptotic cells (Fig. 5b), while ectopic overexpression of HMGB1/RICTOR 3′UTR impeded the response to anti-PD-L1 immunotherapy, which expressed as a decrease in the number of apoptotic cells when compared with the negative control group. More importantly, in vivo immunotherapy experiments were conducted using NCG mice were implanted with HMGB1/ RICTOR-manipulated HCC cell lines. We found that ectopic overexpression of HMGB1/RICTOR 3′UTR impeded the response to anti-PD-L1 immunotherapy in vivo (Supplementary Fig. 4b). Taken together, these results indicated that HMGB1 and RICTOR mRNAs can influence immunotherapy outcomes in HCC.

Exosomes encapsulate bioactive molecules and transmit intracellular cargo to affect the extracellular environment and the immune system^19,20^. The interaction between PD-L1 on the cancer cell surface and PD-1 on T cells results in PD-L1 mediated-tumor immune escape. Intriguingly, it was recently demonstrated that PD-L1^+^ exosomes suppress immune cell cytotoxicity to permit tumor growth similar to PD-L1 expression on the tumor cell surface, and PD-L1^+^ exosomes appear promising predictors of clinical immunotherapy responses in patients^21^. In this study, transmission electron microscope revealed that purified exosomes exhibited cup- or sphere-shaped morphology with double-membrane structures (Supplementary Fig. 4c), similar to previously described exosomes. Flow cytometry showed the enrichment of exosomes marker CD63 in cell-derived exosomes (Supplementary Fig. 4d). Note that, flow cytometry analysis indicated a higher proportion of PD-L1^+^ exosomes released by liver cells that overexpress HMGB1/RICTOR 3′UTR (Fig. 5c), while the HMGB1/RICTOR interference HCC cell lines produced significantly fewer PD-L1^+^ exosomes (Fig. 5c). In addition, another experimental method such as Western Blot was also used to validate the results of flow cytometry analysis (Supplementary Fig. 4e).

Taken together, these results indicated that HMGB1 and RICTOR mRNAs may epigenetically impede HCC responses to anti-PD-L1 immunotherapy through upregulation of PD-L1^+^ exosomes activity.

### Epigenetic crosstalk between HMGB1 with RICTOR regulates glutamine metabolism and PD-L1 expression through mTOR signaling

**Fig. 6:**
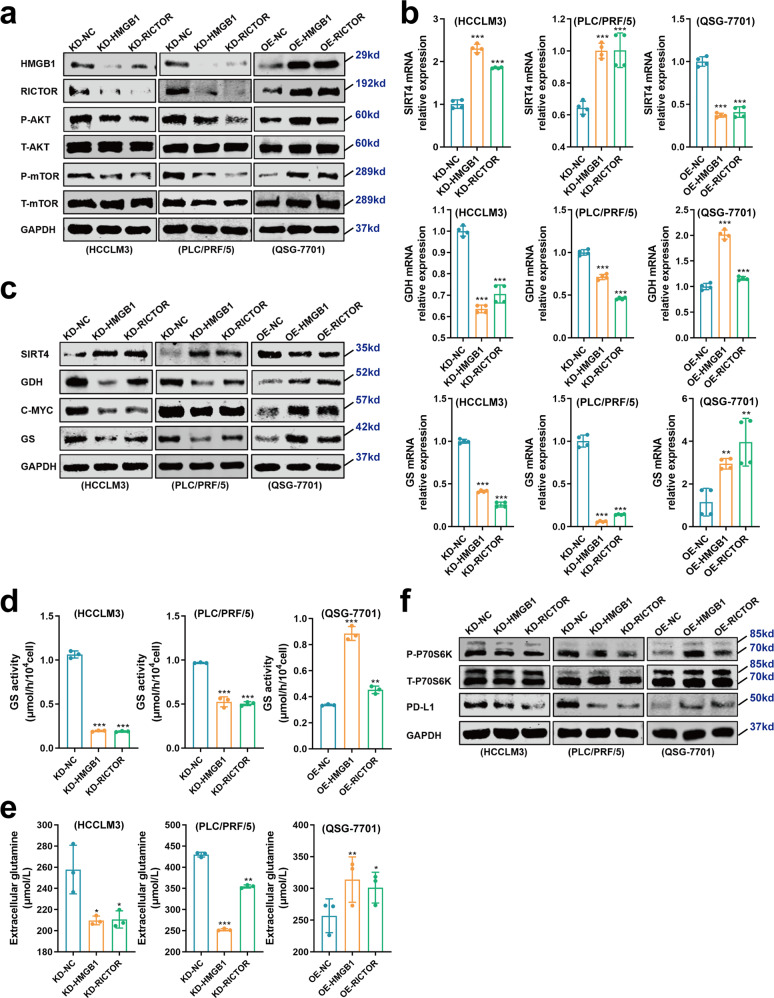
Glutamine metabolism and PD-L1 expression are regulated by the HMGB1–RICTOR epigenetic crosstalk effects on the mTOR pathway. **a** Western blot showing the levels of AKT and mTOR phosphorylation in treated HCC cell lines. **b**, **c** mRNA (**b**) and protein (**c**) levels of enzymes related to glutamine metabolism were determined by qRT-PCR and Western blot, respectively. **p < 0.01, ***p < 0.001. **d** GS activity was measured using a colorimetric kit in indicated HCC cells. **p < 0.01, ***p < 0.001. **e** Glutamine concentrations in cell culture supernatants from treated HCC cells were determined. *p < 0.05, **p < 0.01, ***p < 0.001. **f** Western blot analysis comparing P70S6K and PD-L1 levels in the indicated HCC cells

Next, we further explored the downstream mechanisms underlying the HMGB1-RICTOR epigenetic crosstalk effects on HCC tumorigenesis and immunotherapy responses. As mentioned above, RICTOR is a key element of the mTORC2 which modulates the activity of the AKT-mTORC1 signaling pathway^22^. We, therefore, first evaluated the activity change in mTORC1 by measuring the AKT activation status. Overexpression of HMGB1/RICTOR 3′UTR remarkably enhanced Ser473 phosphorylation of AKT thus elevating the activity of the mTORC1 signaling pathway (Fig. 6a). Conversely, interference of HMGB1/RICTOR led to decreased AKT activation in HCC cells and dampening of AKT-mTORC1 signaling (Fig. 6a).

Glutamine metabolism is well-known to play a crucial role in HCC development as one of the key factors affected during metabolic reprogramming^23–25^. Central enzymes within the glutamine metabolism pathway have been previously linked to mTOR signaling. For example, glutamine synthetase (GS) can be stimulated by the induction of C-MYC via the mTORC2-AKT axis^25,26^. Moreover, glutamate dehydrogenase (GDH) can be activated by de-repressing its negative regulator, SIRT4, at the transcriptional level via mTORC1 dependent signaling^27^. CCK8 assay was performed to detect cell growth after the interference of SIRT4, C-MYC, GS, or GDH in HCCLM3, PLC/PRF/5, and QSG-7701 cell lines using siRNA (Supplementary Fig. 5a). As excepted, the inhibition of C-MYC, GS, or GDH in HCC cell lines led to a significant blockage of malignant cell proliferation, while the downregulation of SIRT4 promoted HCC cell growth (Supplementary Fig. 5b). It is indicated the tumor-promoting effect of C-MYC, GS, and GDH and tumor-inhibiting function of SIRT4.

Given our finding that HMGB1 epigenetically crosstalk with RICTOR contributed to the activation of mTOR signaling, we examined the activity of key molecules associated with glutamine metabolism in the HMGB1/RICTOR-manipulated HCC cells. We found that overexpression of HMGB1/RICTOR 3′UTR downregulated SIRT4, thus de-repressed the expression of GDH (Fig. 6b, c), and upregulated C-MYC together with GS (Fig. 6b, c). Furthermore, GS activity (Fig. 6d) and the concentration of extracellular glutamine (Fig. 6e) were significantly elevated in the HMGB1/RICTOR 3′UTR overexpressing cells. In contrast, the opposite phenotypes were observed after the interference of HMGB1/RICTOR (Fig. 6a–e). Together these data indicated that epigenetic crosstalk between HMGB1 and RICTOR can stimulate glutamine metabolism via mTOR signaling.

As expected, the cells transfected with sites-muted HMGB1/RICTOR 3′UTR plasmids (OE-HMGB1/RICTOR 3′UTR^MUT^ groups) showed insignificant activation of AKT-mTOR signaling pathway and glutamine metabolism (Supplementary Fig. 6a, b). These results indicated that HMGB1 and RICTOR mRNAs epigenetically promote the activation of the AKT-mTOR signaling pathway and glutamine metabolism in HCC depending on the miR-200 family binding sites in 3′UTR.

Finally, previous evidence from lung cancer has shown that activation of the AKT-mTORC1-P70S6K pathway can upregulate PD-L1 expression levels^28^. We, therefore, considered whether this regulatory axis may be active in the liver cancer context. Notably, Western blot assays showed that ectopic overexpression of HMGB1/RICTOR 3′UTR promoted the activity of P70S6K and further upregulated the expression of PD-L1 in the QSG-7701 cell line (Fig. 6f). To further clarify the effect of mTORC1 signaling, we treated HMGB1/RICTOR 3′UTR overexpressed HCC cells (OE-HMGB1/RICTOR groups) with Rapamycin (a mTORC1 specific inhibitor) and estimated the PD-L1 levels. We found that the elevated proportions of PD-L1^+^ exosomes in HMGB1/RICTOR 3′UTR overexpressed HCC cells were downregulated after inhibition of mTORC1 (Supplementary Fig. 6c), which confirmed that HMGB1 and RICTOR mRNAs epigenetically promotes PD-L1 level in HCC cells via an mTORC1-dependent mechanism. On this basis, it is reasonable to speculate that the increased expression of PD-L1 resulting from the effects of HMGB1–RICTOR epigenetic crosstalk contributes to the increased production of PD-L1^+^ exosomes.

Moreover, we confirmed our conclusion in the in vivo study. Downregulation of HMGB1/RICTOR mRNAs significantly inhibited phosphorylation of AKT and mTOR, and expressions of GS and GDH in subcutaneously implanted tumors (Supplementary Fig. 7a, b). Conversely, ectopic overexpression of HMGB1/RICTOR 3’UTR led to opposite effects in the xenograft models (Supplementary Fig. 7a, b). Also, downregulation of HMGB1/RICTOR mRNAs significantly inhibited PD-L1 expressions in the subcutaneously implanted tumors (Supplementary Fig. 7c). In contrast, ectopic overexpression of HMGB1/RICTOR 3′UTR led to opposite effects in the xenograft models (Supplementary Fig. 7c). These results were in line with those in vitro findings and solidified the conclusion that epigenetic crosstalk between HMGB1 and RICTOR can stimulate glutamine metabolism and promote PD-L1 expression via mTOR signaling in HCC.

## Discussion

The limited early therapeutic rates of HCC represent an unmet and urgent need. In this study, we focused our efforts on understanding whether the liver disease-related gene, HMGB1, participated in epigenetic regulatory mechanisms beyond its potential as a protein-coding gene. We systematically combined bioinformatic and expression analyses to uncover the candidate crosstalk ceRNA, RICTOR, which acted specifically in HBV^+^ early stage HCC. Furthermore, we showed that HMGB1 regulates the expression of RICTOR in HCC cells in an “RNA–RNA” crosstalk manner. For example, overexpression of HMGB1 3′UTR increased the expression level of RICTOR at both the RNA and protein levels.

From our previous studies, we established the significance of the protein-coding function of HMGB1 in liver diseases including NAFLD and HCC^12–15^. Herein, through an integrated analysis of gene expression, reporter gene assays, and FISH, we demonstrated that HMGB1 mRNA interacts with RICTOR mRNA by competitively binding to the miR-200 family, especially miR-429. In line with its high expression levels in HBV^+^ early stage HCC, HMGB1, and RICTOR mRNAs were functionally associated with the stemness properties of HCC cells. Overexpression of HMGB1 and RICTOR 3′UTR in liver cell lines remarkably promoted their malignant proliferation, self-renewal, and tumorigenesis. Notably, our results define a novel epigenetic regulatory mechanism in HCC together with highlighting how a single-RNA species can contribute to both “protein-to-protein” signaling and “RNA-to-RNA” crosstalk networks. Currently, the efficacy of targeted drug therapy for HCC is generally unsatisfactory. One of the reasons may be that current drugs mainly direct at protein targets generated by coding genes, without taking into account the hidden “RNA–RNA” crosstalk network. These findings, therefore, provide new insights into a potential therapeutic target of epigenetic-regulatory mRNAs in early stage HCC.

**Fig. 7:**
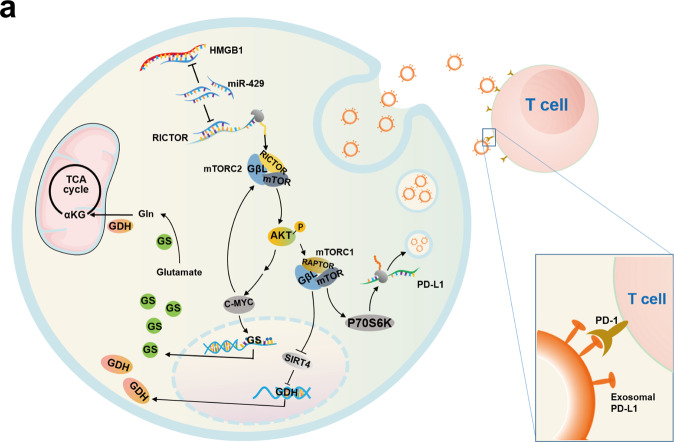
Mechanism atlas. **a** A model of the RNA–RNA crosstalk network involving HMGB1 and RICTOR acting in the early stage HCC

Glutamine is regarded as both a substrate source for the TCA cycle and a regulator of redox homeostasis, fulfilling a vital role in cancer cell adaption and survival^27^. Mechanistically, we demonstrated that epigenetic crosstalk between HMGB1 and RICTOR provides dual effects on glutamine metabolism. First, there was increased glutamine anabolism as shown by the activated mTORC2-AKT axis promoting GS expression via C-MYC induction. Interestingly, considering C-MYC can, in turn, activate the mTORC2-AKT cascade^16^, the promotion of GS through the mTORC2-AKT axis through C-MYC may constitute a positive feedback loop (Fig. 7). Secondly, activating mTORC1 signaling, resulted in the ablation of SIRT4, abrogating its downstream repression of GDH. Notably, GDH is the key enzyme that catalyzes the final reaction of glutamine catabolism to supplement the TCA cycle with glutamine-derived metabolites. Together these alterations were shown to promote the initiation and development of HCC.

It has been demonstrated that adaptive immunity actively prevents HCC tumorigenesis. Indeed, pre-cancerous liver tissue exhibits PD-L1 accumulation, and PD-L1 blockade results in tumor regression in the early stages of HCC^29^. Nevertheless, from the perspective of immunotherapy, both PD-L1 pathological positive and negative patients exhibit unsatisfactory response rates of only 10–30%^30^. However, if predictors of immunotherapy responsiveness could be identified, it has been estimated that the median overall survival of eligible HCC patients would improve by at least two years compared to traditional systemic therapy^31^. In this study, we confirmed the aberrant expression of PD-L1 in the early stage HCC, thus laying the theoretical foundation for anti-PD-L1 administration in it. Mechanistically, an “RNA–RNA” crosstalk network involving HMGB1 and RICTOR upregulated intracellular PD-L1 levels by epigenetically activating the AKT-mTORC1-P70S6K signaling pathway. This process may facilitate increased PD-L1 encapsulation into exosomes and as previously shown, PD-L1^+^ exosomes can suppress immune cell cytotoxicity^21^, proposing that responses to anti-PD-L1 immunotherapy would be enhanced in HCC cells by inhibiting the epigenetic crosstalk of HMGB1 with RICTOR. Therefore, the expression of HMGB1 and RICTOR could be envisioned as proxy indicators of increased PD-L1^+^ exosomes and be used to guide therapeutic decision-making in HCC.

In summary, we deconstructed the details of an HMGB1-driven “RNA–RNA” crosstalk network which acts specifically in HBV^+^ early stage HCC (Fig. 7). HMGB1 epigenetically regulates RICTOR expression through ceRNA-mediated regulation, competitively binding to the miR-200 family, especially miR-429. The HMGB1–RICTOR “RNA–RNA” crosstalk network facilitates glutamine metabolism in HCC cells via activating a positive feedback loop involving mTORC2-AKT-C-MYC to upregulate GS expression, also inducing mTORC1 signaling to derepress SIRT4 effects on GDH expression. This dual-faceted regulation then enhances the stemness characteristics and tumorigenic capacity of HCC cells. Moreover, this regulatory network impedes the efficacy of immunotherapy through mTORC1-P70S6K-dependent PD-L1 production and PD-L1^+^exosome activity. Collectively, our study highlights the non-coding regulatory role of HMGB1 and defines it as a new HCC therapeutic target and indicator for anti-PD-L1 immunotherapy.

## Materials and methods

### HCC patients and clinical samples

HCC tissues and matched non-tumorous adjacent tissues were obtained from patients who were diagnosed and received surgical resection at the Eastern Hepatobiliary Surgery Hospital, Shanghai, China. No patients received any preoperative anticancer treatment. Patients were recruited from 2006 to 2015. HCC staging was determined by the Barcelona Clinic Liver Cancer staging system (BCLC). All research complied with the principles of the Declaration of Helsinki. Patient samples were obtained following informed consent according to an established protocol approved by the Ethics Committee of Eastern Hepatobiliary Surgery Hospital.

### Animals

The nude mice were housed in individual microisolator cages with free access to sterile water and irradiated normal food in a specific pathogen-free facility. Experiments were conducted by following the criteria outlined in the Guide for the Care and Use of Laboratory Animals, prepared by the National Academy of Sciences and published by the National Institutes of Health (NIH publication 86-23; revised1985). All the animal care protocols and experiments were reviewed and approved by the Animal Care and Use Committee of the Laboratory Animal Research Center at the Eastern Hepatobiliary Surgery Institute, Second Military Medical University.

### In vivo xenograft experiments

Male nude mice (4–6 weeks old, *n* = 5) were housed and fed in standard pathogen-free conditions. A total of 1 × 10^4^ HMGB1/RICTOR 3′UTR ectopic overexpressed and HMGB1/RICTOR interference HCC cells were injected subcutaneously into the flanks of mice, respectively. The subcutaneous tumors were harvested 48 days after injection. Tumor volume was calculated as below: *V* (mm^3^) = width^2^ (mm^2^) × length (mm)/2.

### Cell lines and cell culture

Normal liver cell lines QSG-7701, liver cancer cell lines HCCLM3 and PLC/PRF/5 were routinely cultured as previously described^10^. Cell lines were maintained at 37 °C in an atmosphere containing 5% CO_2_ in DMEM supplemented with 10% fetal bovine serum (GIBCO, Carlsbad, CA, USA). Cells were passed every 1–2 d to maintain logarithmic growth. According to the expression of HMGB1 and RICTOR in liver cell lines, we chose the lower HMGB1 and RICTOR-expressing normal liver cell line QSG-7701, to perform the gain-of-function experiments. The higher HMGB1 and RICTOR-expressing HCC cell line HCCLM3 and PLC/PRF/5 were used to perform the loss-of-function experiments.

### Fluorescence in situ hybridization

The frozen sections were examined based on in situ hybridization using digoxin-labeled HMGB1, RICTOR, and miR-429 probes and digoxin detection kit (Boster biological technology, Guangzhou, China) according to the suggested procedures. The in situ hybridization signals were detected and analyzed with a fluorescence confocal microscope (Leika SP8, Germany). For each channel, all images were acquired with the same settings.

### Isolation of PBMCs from whole blood

The whole blood of HCC patients was obtained from patients who were diagnosed and received surgical resection at the Eastern Hepatobiliary Surgery Hospital, Shanghai, China. PBMCs were obtained by density gradient centrifugation on Ficoll–Paque Premium (Stem Cell Technologies, Canada) following the manufacturer’s protocol. Briefly, the buffy coat was diluted 3-fold with sterile PBS, and 35 mL of this suspension was slowly poured over a 15 mL separation medium. After 30 min of centrifugation at 1700 rpm with brakes turned off, the interphase was moved to a new reaction tube, washed twice with PBS, and centrifuged for 10 min at 1500 rpm. The resulting cell pellets were suspended in a complete RPMI-1640 medium and were seeded into a six-well plate. PMBCs were stimulated by cytokine IL-2 with a final concentration of 10 ng/μl for 48 h before use.

### PBMC-mediated killing assay and atezolizumab treatment assay in vitro

Totally, 2 × 10^5^ adherent HCC cells were seeded into a 6-well plate and incubated for 12 h. Then, stimulated PMBCs were centrifuged at 400×*g* and suspended with a complete RPMI-1640 medium. PBMCs were counted and added into HCC cells at an effector to target ratio of 8:1 in each well of a 6-well plate, and treated with vehicle or atezolizumab (10 μg/mL). After treatment for 48 h, media were removed from wells, including suspended PBMCs and tumor cells. The remaining attached tumor cells were washed thrice with a culture medium to flush any non-adherent cells remaining in the wells. The assay was done in triplicate.

### Statistical analysis

All statistical analyses were performed with SPSS 18.0 software. Qualitative variables were analyzed by chi-square test or fisher’s exact test. For continuous variables, if they obey the normal distribution, the Student *t*-test is used to compare the differences. Otherwise, variables were compared using the nonparametric test for which with an abnormal distribution. Differences between groups were compared using analysis of variance when applicable or a nonparametric test. Correlation analysis was performed using the Pearson correlation coefficient method. Unless otherwise specified, the results are presented as the means ± standard deviation. All statistical tests were 2 sided, and *P* < 0.05 was considered statistically significant.

## Supplementary information

Supplementary materials

## Data Availability

All data are available within the article, supplementary materials, or available from the corresponding author upon reasonable request.
