# Supplementary material for: An RNA–RNA crosstalk network involving HMGB1 and RICTOR facilitates hepatocellular carcinoma tumorigenesis by promoting glutamine metabolism and impedes immunotherapy by PD-L1+ exosomes activity
Source: Signal Transduct Target Ther. 2021 Dec 17;6:421. doi: 10.1038/s41392-021-00801-2 (PMC8677721; doi:10.1038/s41392-021-00801-2)
Supplement: Supplementary file 1 — Supplementary materials [file 41392_2021_801_MOESM1_ESM.pdf]

Supplementary Materials for

An RNA-RNA crosstalk network involving HMGB1 and RICTOR facilitates hepatocellular carcinoma tumorigenesis by promoting glutamine metabolism and impedes immunotherapy by PD-L1^{+} exosomes activity

Yanping Wei, Xuewu Tang, Yibin Ren, Yun Yang, Fengliang Song, Jingbo Fu, Shuowu Liu, Miao Yu, Jing Chen, Suyang Wang, Kecheng Zhang, Yexiong Tan, Zhipeng Han, Lixin Wei, Baohua Zhang, Zhangjun Cheng, Liang Li, Hongyang Wang

Correspondence to hywangk@vip.sina.com

This PDF file includes:

Materials and Methods

Supplemental Reference

Supplementary Figures 1-7

Materials and Methods

CCK8 and spheroid formation assay

CCK8: The viability of HCC cells was determined by Cell Counting Kit 8 (Dojindo, Japan) and measured at OD450 nm with the BioTek Gen5 system (BioTeck, USA). Spheroid formation: HCC cells were seeded in 6-well low-adhesion plates at a density of 3×10^{3} cells per well. After 3w, spheroids were observed using Image J software (NIH Image).

Lentiviral Infection

The lentivirus for over-expression and interference of HMGB1/RICTOR 3'-UTR were designed and produced by the Heyuan Biotech Company (Shanghai, China). For HMGB1/RICTOR 3'-UTR stable overexpression system, H145 pLenti-EF1a-EGFP-F2A-Puro-CMV-MCS vector was used for constructs. Component sequence: pLenti-EF1a-EGFP-F2A-Puro-CMV-HMGB1 (or RICTOR) 3'UTR. Cloning site: EcoR I/BamH I. The sequence of 3'UTR of HMGB1 and RICTOR are as following. (Green indicated the binding sites of miR-200a, and red indicated the binding sites of miR-200b/429) :

HMGB1 3'UTR :

GTTGGTTCTAGCGCAGTTTTTTTTTTCTTGTCTATAAAGCATTTAACCCCCCTGTAC
ACAACTCACTCCTTTTAAAGAAAAAAATTGAAATGTAAGGCTGTGTAAGATTTGT
TTTTAAACTGTACAGTGTCTTTTTTTGTATAGTTAACACACTACCGAATGTGTCTT
TAGATAGCCCTGTCCTGGTGGTATTTTCAATAGCCACTAACCTTGCCTGGTACAGT
ATGGGGGTTGTAAATTGGCATGGAAATTTAAAGCAGGTTCTTGTTGGTGCACAGC
ACAAATTAGTTATATATGGGGATGGTAGTTTTTCATCTTCAGTTGTCTCTGATGC
AGCTTATACGAAATAATTGTTGTTCTGTTAACTGAATACCACTCTGTAATTGCAAA
AAAAAAAAAAAAGTTGCAGCTGTTTTGTTGACATTCTGAATGCTTCTAAGTAAAT
ACAATTTTTTTTATTAGTATTGTTGTCCTTTTCATAGGTCTGAAATTTTTCTTCTTG
AGGGGAAGCTAGTCTTTTGCTTTTGCCCATTTTGAATCACATGAATTATTACAGTG
TTTATCCTTTCATATAGTTAGCTAATAAAAAGCTTTTGTCTACACACCCTGCATAT
CATAATGGGGGTAAAGTTAAGTTGAGATAGTTTTCATCCATAACTGAACATCCAA

AATCTTGATCAGTTAAGAAATTTCACATAGCCCACTTACATTTACAAACTGAAGA

GTAATCAATCTACTCAAAGCATGGGATTATTAGAATCAAACATTTTGAAAGTCTG

TCCTTGAAGGACTAATAGAAAAGTATGTTCTAACCTTTACATGAGGACTCTATTC

TTTAACTCCCATTACCATGTAATGGCAGTTATATTTTGCAGTTCCCACATTAAAGA

AGACCTGAGAATGTATCCCCAAAAGCGTGAGCTTAAAATACAAGACTGCCATATT

AAATTTTTTGTTGACATTAGTCTCAGTGAAGACTATGAAAATGCTGGCTATAGAT

GTCTTTTCCCATTTATCTAAATATGGACTGCTCAGGAAACGAGACTTTCCATTACA

AGTATTTTTAATTAATTGGGCCAGCTTTTCAAACAAAGATGCCACATTCAAAATA

GGGTATATTTTCCTATATTACGGTTTGCCCCTTTATAAATCCAAGTAGATAGGAAG

AAAGAAGACAAACTTTGCATCTCAGTATGAATTATTCAATTTATTTGAATGATTTT

TCTTTACAAAACAAACTCATTCATTAGTCATGTTTATCTGCTTAGGAGTTTAGGGA

ACAATTTGGCAATTTTGTGGTTTTCGAGATTATCGTTTTCTTAAAGTGCCAGTATT

TTAAAATAGCGTTCTTGTAATTTTACACGCTTTTGTGATGGAGTGCTGTTTTGTTA

TATAATTTAGACTTGGATTCTTTCCATTTGCATTTGTTTATGTAATTTCAGGAGGA

ATACTGAACATCTGAGTCCTGGATGATACTAATAAACTAATAATTGCAGAGGTTT

TAAATACTAGTTAAATGGCTTTCACTTAAGAACTTAAGATTTTGTTACATATTTTT

AAATCTTGTTTCTAATAATACCTCTTAGCAGTACCTTTTAAATAAGTATAAGGGAT

GGCAAAGTTTTTCCCTTTAAAAATACTCACTTTATGCTTATAAATAGGTTAATGGG

CTGATAAAAGGTTTTGTCAAACATTGCAAGTATTCGGTGCTATATATAAAGGAGG

AAAAACTAGTTTTACTTTCAGAATGATTTAAACAAGATTTTTAAAAACAAGATAC

ATGCAAGCGAACAGCAGGGTTAGTGATAGGCTGCAATTGTGTCGAACATCAGAT

TTTTTGTTAAGAGGAGCAAATGACTCAATCTGATTTAGATGGAAGTTTCTACTGT

ATAGAAATCACCATTAATCACCAACATTAATAATTCTGATCCATTTAAAATGAAT

TCTGGCTCAAGGAGAATTTGTAACTTTAGTAGGTACGTCATGACAACTACCATTT

TTTTAAGATGTTGAGAATGGGAACAGTTTTTTTAGGGTTTATTCTTGACCACAGAT

CTTAAGAAAATGGACAAAACCCCTCTTCAATCTGAAGATTAGTATGGTTTGGTGT

TCTAACAGTATCCCCTAGAAGTTGGATGTCTAAAACTCAAGTAAATGGAAGTGGG

AGGCAATTTAGATAAGTGTAAAGCCTTGTAACTGAAGATGATTTTTTTTAGAAAG

TGTATAGAAACTATTTTAATGCCAAGATAGTTACAGTGCTGTGGGGTTTAAAGAC

TTTGTTGACATCAAGAAAAGACTAAATCTATAATTAATTGGGCCAACTTTTAAAA

TGAAGATGCTTTTTAAAACTAATGAACTAAGATGTATAAATCTTAGTTTTTTTGTA
TTTTAAAGATAGGCATATGGCATATTGATTAACGAGTCAAATTTCCTAACTTTGCT
GTGCAAAGGTTGAGAGCTATTGCTGATTAGTTACCACAGTTCTGATGATCGTCCC
ATCACAGTGTTGTTAATGTTTGCTGTATTTATTAATTTTCTTAAAGTGAAATCTGA
AAAATGAAATTTGTGTGTCCTGTGTACCCGAGGGGTAATGATTAAATGATAAAGA
TAAGAAAA

RICTOR 3’UTR:

AGTTAGTATTGGCCTGAATGTTTTGTGTTTTTTTTTTTGTTTTTTTTTTTTACTGTTA
CTTTGAAAAATATGTATGTATACCTTATCATATCTGCCTATATCACTTACTTTGGG
GAGATACTCAGAGCTTTGTGGTTATCAGTATACTAAAAAAAAAAAAAAGTCTAC
GCTTAAATTTATAGTGCTATTTGGTTTCTCCATGATTTCACTGACAGGTCTAATAC
ATTTTCTTTGAGTACTTGTTTGTAAAAAGTAGACTTTATGGTGAAAAATACATGCA
GTGCCAAGTGATTAACTTAAGTGTTTAAAAATATTAAATTATAGCAGAAGAGGTT
AGGAATGATATCAGCAGTAATAGAAATAATTGAGAAAATCATCTATAAATAATA
GATATTACAGACTATAGAATACCAAAATAATGTCAATACTGTAGTTTTTAAAGAT
TTTAGGATTAATCTTAGTCCATATAAATTTGTACTATTGGTAATTATTGAATAATT
GGGAGGAATCTGGGCAGTTGTGCTGGTTGTAAACTATGAATTTCTAATCGTAAAG
TGAATTGTTATTTCTAATTGAACTTTTTTTCAAGAACAGATTTCAGCCTCACATAC
TAAGTAAATACTGATAAATAAGGAAATTAGAAATTTAGTATTCATAATTAAATAT
GCTCTAAAATTTCCTATACTTTTATTTCCTGTTTATTCTTAGGTAGATTGGAAGGG
GGAAACAGTCTGTTCTCCCTAATTAAATTTTTTCTAATAACGATTAGTAGAATATG
GACATTCTATATGACAGTGACATTAAAAGAGGCTCTTTGGAAGTATATACATTAT
TAACATAATGTGTACAAGTCCTTTTGAAATGACAACTTTAATGGGTTTCAGCTCTT
TTATCTAGAGCTTGAGATAATTCAAGCTGAGTTTTTCAGGGCATATCACAACGGC
CAAGTGTTCAGCAGTGGGATATCAATGCTTATTTACATTTTCCTACTGCTATTTAT
ATAAAATGTTATTCCATTCAGAGGATGCCTTTTATCCCCACATTAAAGCACAGAT
CATTAAGCAATAAAAACCAAATTGTCTGTCATTCAAATTATAACTGCAGTTATTTT
TGCATGGTAAGAGTGAGGTGCTAATTTTGTGTGAGATGAACTTTGTAAACTACTT
TGGGAAATGTTCTTTGGAAGTAAGGTTTTTTCTCCTTTAGTCTTATGCTTCCACTTT
TGTCTCAGATTCACAATCCATTAAAACATGGGGAAAAAAGAAAAGGTAAAATTG

AGAGACTTTTGTTAGAGGAGCTATTTGGAATGAACCAACATTTCAGATTTTCCAA
AATGTAAGTTAGGAAGTCTCCATTGTCTCTGCATTAACAAAATACACTGTTACTA
TCTTAATCTCAAGAGTGTCATTACAGTGAGAATCTCATTTAAAAGCATACCAGTG
AAATTAATAGCAGTGCTTATCAAAGAACACTGAAATCTGTGAGAATCTTTCTAGG
AGCATTCTTTTCTTCTTTTAGTTCCAAGTTCCAGGGTATTTTTCATTCCTAGTAGGT
TTATATGACTCACAGAATGTGGACTTTTTTCCTGTTTGGAGTATTTTTGTAATGTA
AGTATCGGATAGCTGCACCACAGCATGCATAAATTGCACATTTTGTTTTACTTTCT
TTATAGAATATTTAATTTCAAAAATATAATTTATGCCAAAAAAAGCATACCTTTC
AATTTTGCTACTTGGTTGATTTAGCACAAAATGCAAAGTCTTGGGGCAGAGAGGG
GGAGTGAAAAAAATTTTATAGGTAATTGTTACAAAAATACCTGTCAGAAACCCTA
AAGCTGCATTGTAAAACAAATGGTGTAAACTAGTTTTGAAAAGTGGTAAGGAATT
GTGAAAAAAATCTCAGACTTAATGCTCTCTAACCACATGAGTTTCTTCTTTTTTAT
TTAGTAATACGCTGCTACATATTTGGAGGTTCTGGTGTTTGTAGGTCACTGAACA
GACATTGAAATCTGATTTATATTGTATAACTGTAACATAGAAAGAAAAAGTATTT
AT

The vector map used for this overexpression constructs is as following, further information about these plasmids can be obtained upon request:

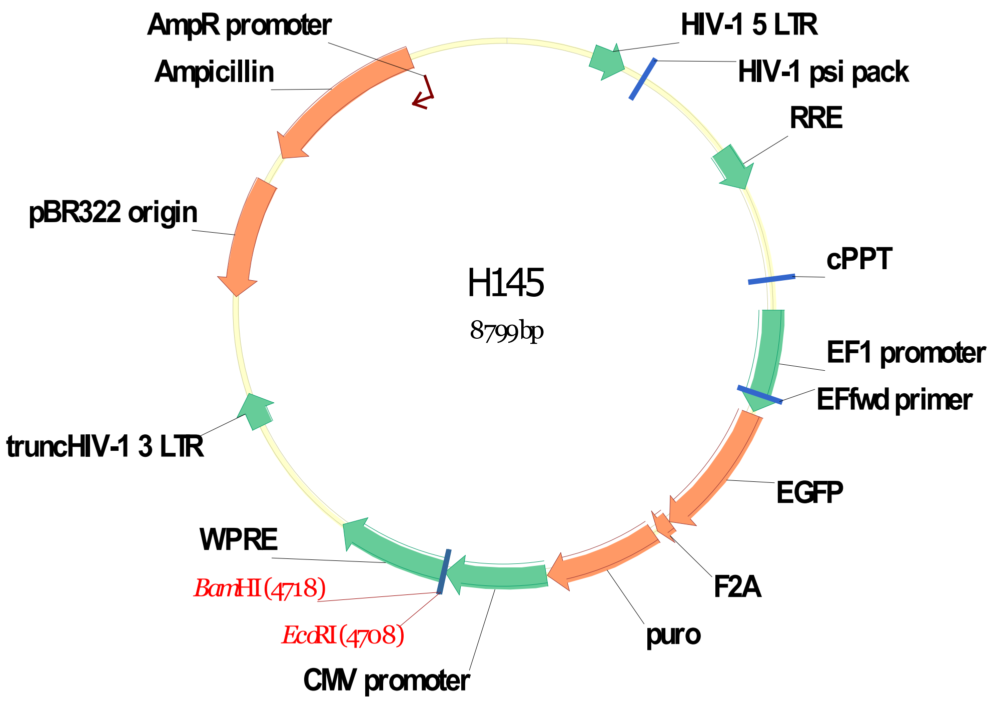

Lentiviral infection was carried out according to the manufacturers' instructions. HCCLM3, PLC/PRF/5 and QSG-7701 cells were infected with the concentrated virus at a multiplicity of infection (MOI) of 20 in the presence of 8 μg/mL polybrene (Sigma, Saint Louis, USA) for 12 hours. Puromycin with the final concentration of 1ng/μl was used to screen infected cells according to the manufacturers' instructions. Expression level of HMGB1/RICTOR 3'UTR in the stably infected cells was validated by qRT-PCR.

Mimics, siRNAs, and plasmids transfection

MiR-200a, miR-200b and miR-429 mimics for *in vitro* transfection, and their respective negative controls were purchased from Ribobio Co. (Guangzhou, China). The siRNAs for SIRT4, C-MYC, GS and GDH were purchased from Biotend Co. (Shanghai, China). Plasmid for over-expression of RICTOR, wild-type and miR-200 family binding sites-mutated HMGB1 and RICTOR 3'UTR were designed and produced by the Heyuan Biotech Company (Shanghai, China). Transfection were performed with JetPRIME reagents (Poly plus, Illkirch, France) according to the manufacturers' instructions. Briefly, for each well (6 well, for example), add 10 μl (20μM storage concentration) mimics/siRNA, or 500ng plasmids into JetPRIME buffer. Mix by pipetting up and down. Then, JetPRIME was added into the mimics/siRNAs/plasmids duplexes, homogenize by vortex immediately for 10 seconds. Incubate for 10 minutes at room temperature to allow transfection complexes to form between duplexes and JetPRIME. Then, add transfection mix into the 2ml cell culture medium to complete a final concentration of 100nM mimics. Finally, homogenize by gently swirling the plate.

Flow cytometry analysis

Flow cytometry was used to analyze the exosomes phenotype derived from cultured HCC cells. Exosomes purified from cell supernatant were co-incubated with biotin-labeled anti-CD63 mAb (353018, Biolegend, San Diego, CA). Next, the streptavidin beads were added and were incubated for 2h at RT. The bead/anti-

CD63Ab/exosome complexes were then co-incubated with the fluorescein-conjugated monoclonal antibodies anti-PD-L1 PE (557924, BD Pharmingen, New Jersey, USA) or with the isotype control (555749, BD Pharmingen, New Jersey, USA) at 4°C in the dark overnight. The samples were analyzed using Beckman MoFlo XDP™ flow cytometer (Beckman Coulter, USA). The analysis of the raw data of exosomes flow cytometry was performed according to the previous study[1]. Percentages of PD-L1^{+} Exo were resulted from the relative fluorescence values gated by isotype controls.

Exosomes identification

The identification of exosomes was performed. Exosomes isolated from cell cultured supernatant of HCCLM3, PLC/PRF/5 and QSG-7701 cells using ultracentrifugation. Exosomes were examined and photographed by Electron Microscopy using negative staining. Exosomes markers were detected using Flow cytometry assays using anti-CD63 antibodies.

Rapamycin treatment

Cell lines (OE-NC/OE-HMGB1/OE-RICTOR cells) were treated with rapamycin at the final concentration of 100nM for 48h.

Luciferase Reporter Assay

A dual-luciferase reporter assay was performed to investigate whether HMGB1 and RICTOR is regulated by miR-200a, miR-200b, and miR-429, respectively. The luciferase reporter gene vector containing either wild type 3'UTR or feasible binding site mutant 3'UTR (3'UTR^{mut}) of target genes was constructed by the Genechem Company (Shanghai, China). HEK-293T cells were cultured in 24-well plates and co-transfected with 500ng/well HMGB1-3' UTR and RICTOR-3' UTR dual-luciferase reporter gene plasmid and 100nM miR-200a, miR-200b, and miR-429 mimics together with jetPRIME reagents (Poly plus). Then luciferase activity was detected by dual-luciferase reporter assay system (Promega) after 48 hours of incubation.

GS activity detection assay

The GS activity of cells was assessed by Glutamine synthetase assay kit (Nanjing Jiancheng Bioengineering Institute) according to manufacturer's protocols. In brief, 10^4 cells were collected for each group. Extracting buffer were added into cell pellet and mixed well, then cells were disrupted by ultrasonication. Add corresponding buffer into tube and incubate for 30 min at 25°C. The supernatant was collected and measured at OD540 nm with the BioTek Gen5 system (BioTeck, USA). GS activity was calculated according to the manufacturer's protocols.

Extracellular glutamine detection assay

The concentration of extracellular glutamine was assessed by Glutamine measurement kit (Nanjing Jiancheng Bioengineering Institute) according to manufacturer's protocols. In brief, $2*10^6$ cells were seeded in 6cm dish for each group and cultured with medium without glutamine at 37°C, 5%CO_2 for 48 hours. The supernatant culture media were collected and mixed well with the testing buffer, and were incubated for 15 min at 37°C. Samples were measured at OD630 nm with the BioTek Gen5 system (BioTeck, USA). Glutamine concentration was calculated according to the manufacturer's protocols.

TUNEL assay

Cell apoptosis was assessed by One-step TUNEL Apoptosis Kit (RiboBio, Guangzhou, China) according to manufacturer's protocols. In brief, cells were fixed with 4% paraformaldehyde after the indicated treatment, followed by incubation with 0.5% Triton X-100 for cell permeabilization. Next, cells were incubated with TdT Enzyme, TdT Buffer, and TAM-dUTP Mixture at 37°C for 2 h in the dark. Apoptotic cells were observed by fluorescence microscope.

In vivo PBMC killing assay

Male NCG mice (NOD-Prkdcem26Cd52Il2rgem26Cd22/NjuCrl) of 4-week-old were purchased from the Nanjing Biomedical Research Institute of Nanjing University.

A total of 5×10^6 HCC cells were injected into the right flank of NCG mice. PBMC from healthy donors were activated and expanded as described above. The day before tumor cell injection, PBMC (i.v. 1×10^7 cells) was adoptively transferred to NCG mice via the tail vein. Atezolizumab were injected via the tail vein every 3 days after tumor cell injection. The subcutaneous tumors were harvested 12 days after injection.

Immunohistochemical Staining

5μm consecutive sections were cut by standard methods and were stained for HMGB1 and RICTOR. All paraffin embedded sections were dewaxed in xylene and rehydrated in graded ethanol solutions. Antigens were retrieved with citrate buffer (0.01 M citric acid: pH 6.0) for 15 min at 100°C in a microwave oven. After treated with 3% H_2O_2 for 10 min to block the endogenous peroxidase, the sections were incubated with 10% fetal calf serum for 30 min to reduce nonspecific binding. The primary antibodies (HMGB1: ab79823; RICTOR: ab70374, abCAM) were applied to the sections at 4°C overnight.

Western Blots

Cell lysates were prepared with IP buffer (Beyotime Biotechnology, China) and centrifuged at 12,000g for 20 min to collect the supernatant. Protein concentrations were determined using the bicinchoninic acid (BCA) assay. Immunoblotting was performed using specific primary antibodies as following: rabbit monoclonal anti-p-AKT (Ser473) (4060S, Cell Signaling Technology), rabbit monoclonal anti-AKT (pan) (4691P, Cell Signaling Technology), rabbit monoclonal anti-p-mTOR (Ser2448) (5536P, Cell Signaling Technology), rabbit monoclonal anti-mTOR (2983P, Cell Signaling Technology), rabbit monoclonal anti-p-P70S6K (Ser371) (9208P, Cell Signaling Technology), rabbit monoclonal anti-P70S6K (9202P, Cell Signaling Technology), rabbit monoclonal anti-C-MYC (13987S, Cell Signaling Technology), rabbit polyclonal anti-Glutamine synthetase (ab49873, abcam), rabbit polyclonal anti-SIRT4 (ab231137, abcam), anti-GLUD1 (GDH) (ABN443, Millipore), rabbit polyclonal anti-PD-L1 (17952-1-AP, proteintech), mouse monoclonal anti-GAPDH

(SC-47724, Santa Cruz). Immunocomplexes were incubated with the fluorescein-conjugated secondary antibody (mouse, C61012-05; rabbit, C80416-08, Li-Cor) and then detected using an Odyssey fluorescence scanner (Li-Cor, Lincoln, NE).

RNA isolation and PCR analysis

Total RNAs were isolated from cells or exosomes using TRIzol Reagent (Invitrogen, USA) following the manufacturer's instructions. The complementary DNA template was prepared using oligo (dT) random primers and Moloney Murine Leukemia Virus reverse transcriptase (Promega, USA) according to the manufacturer's protocol. After the reverse transcription reaction, the complementary DNA template was quantitated using real-time PCR technology. The primers used in this study were designed using Primer-BLAST tool provided by www.ncbi.nlm.nih.gov. cDNA was amplified in reaction mix of SYBR Green (Takara, Japan) with LightCycler® 96 Real-Time PCR System (Roche, Basel, Switzerland) according to the manufacturer's instructions. The mRNA levels were normalized against β-actin in cell and tissue lysates. Real-time PCR experiments for each gene were performed on 3 separate occasions.

Supplemental Reference

1. Theodoraki, M.N., Yerneni, S.S., Hoffmann, T.K., Gooding, W.E. & Whiteside, T.L. Clinical Significance of PD-L1(+) Exosomes in Plasma of Head and Neck Cancer Patients. *Clin Cancer Res* **24**, 896-905 (2018).

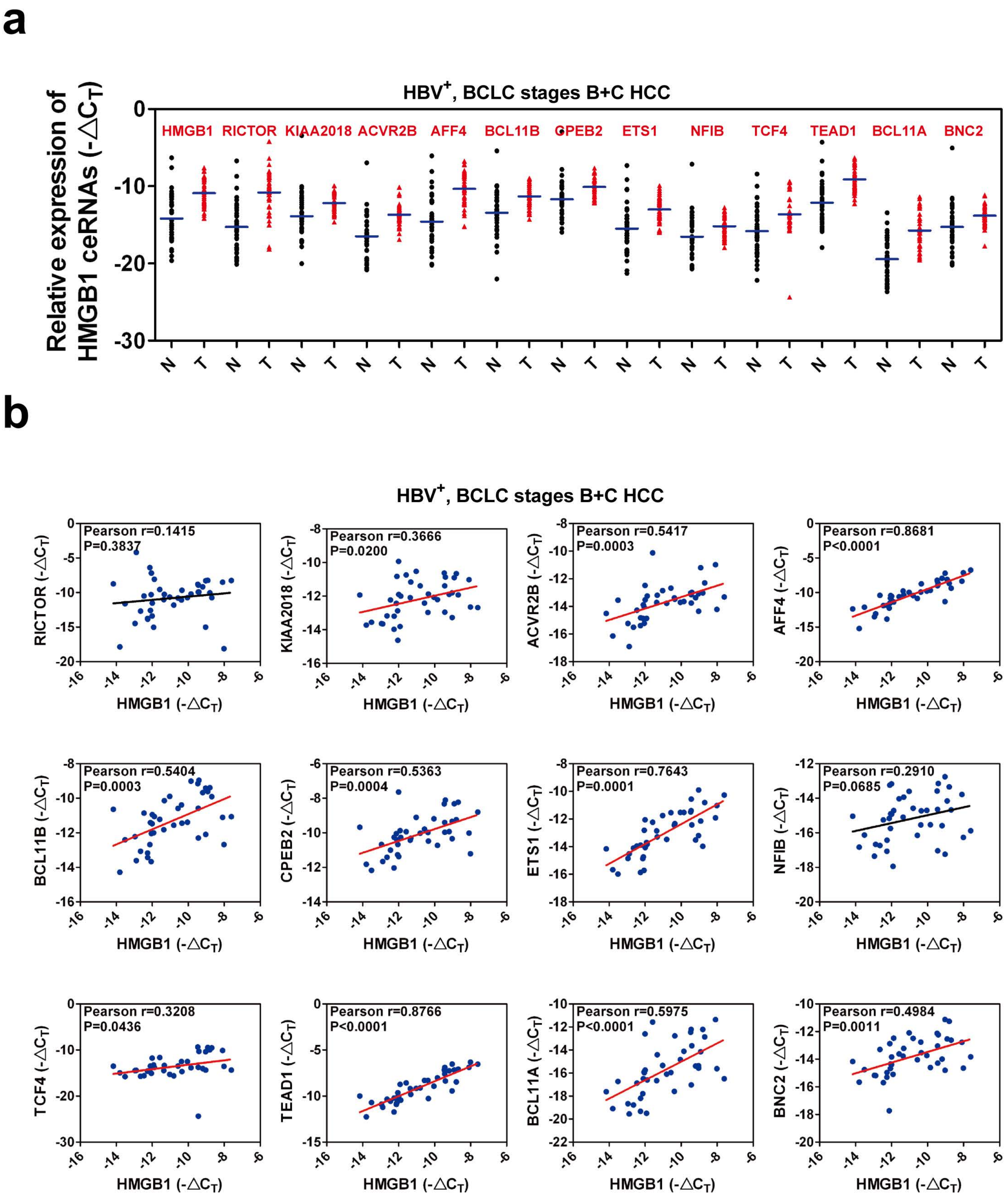

Supplementary Figure 1

Supplementary figure 1. Expression analysis of HMGB1 and candidate ceRNAs.

(a) The RNA expression levels of HMGB1 and the top 12 candidate ceRNAs were validated in an independent HBV^+ BCLC stages B+C HCC sample set by qRT-PCR. n=40. (b) Pearson correlation coefficient analysis between expression levels of HMGB1 and the top 12 candidate ceRNAs, respectively, in HBV^+ BCLC stages B+C HCC tumor tissue. n=40.

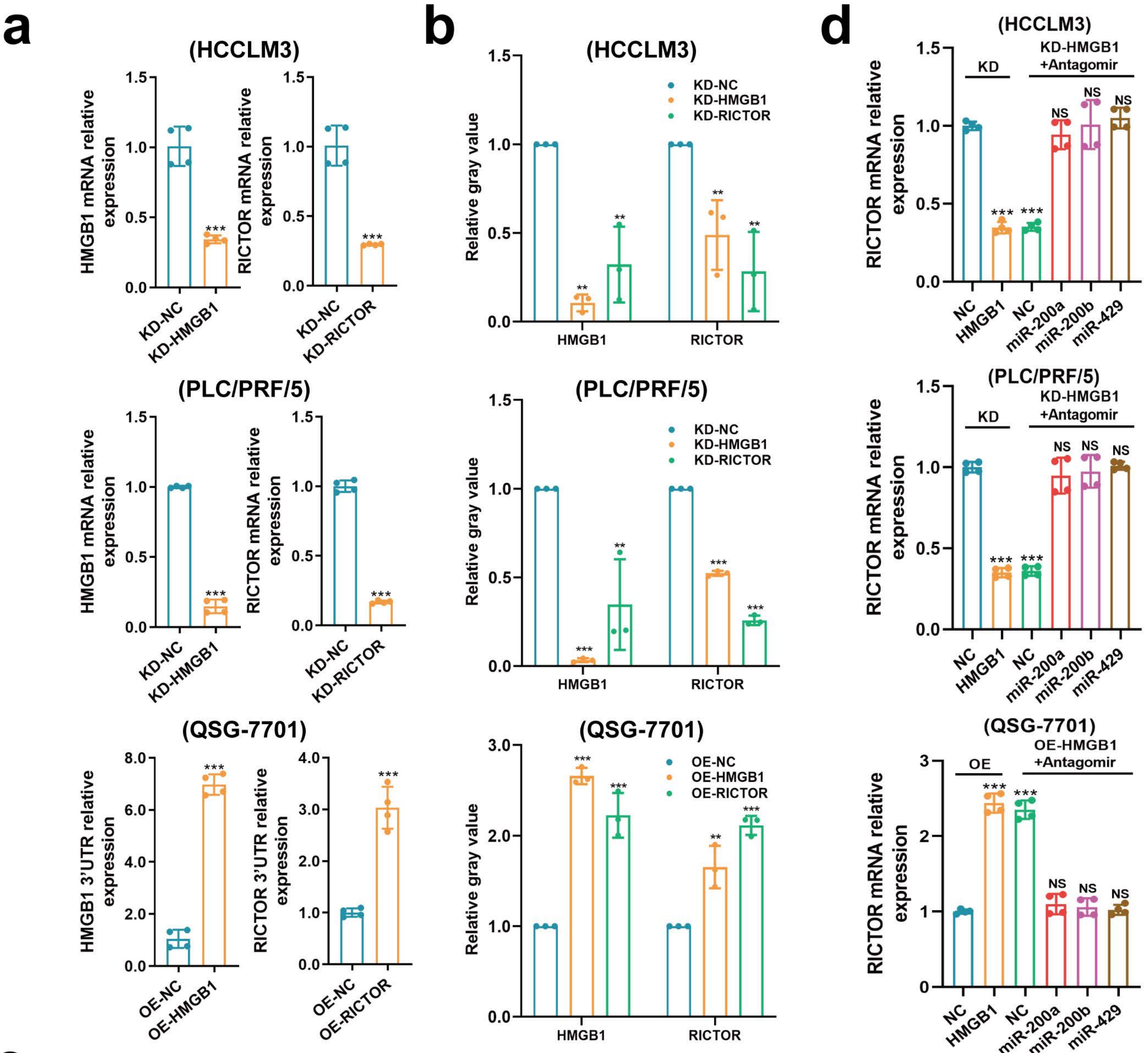

c

name	geneName	targetScanSites	picTarSites	RNA22Sites	PITASites	miRandaSites	CancerNum
hsa-miR-200b-3p	RICTOR	0[0,0]	0[0,0]	0[0,0]	0[0,0]	1[2,48]	3
hsa-miR-200a-3p	RICTOR	0[0,0]	0[0,0]	0[0,0]	1[9,240]	1[9,240]	4
hsa-miR-186-5p	RICTOR	0[0,0]	0[0,0]	0[0,0]	0[0,0]	2[4,38]	3
hsa-miR-137	RICTOR	1[2,141]	3[4,265]	0[0,0]	1[2,141]	1[2,141]	1
hsa-miR-9-5p	RICTOR	0[0,0]	0[0,0]	1[5,201]	0[0,0]	0[0,0]	3
hsa-miR-488-3p	RICTOR	0[0,0]	0[0,0]	0[0,0]	1[14,244]	1[15,252]	0
hsa-miR-181b-5p	RICTOR	0[0,0]	0[0,0]	0[0,0]	0[0,0]	1[2,293]	1

name	geneName	targetScanSites	picTarSites	RNA22Sites	PITASites	miRandaSites	CancerNum
hsa-miR-200b-3p	HMGB1	0[0,0]	0[0,0]	0[0,0]	1[1,5]	2[7,128]	1
hsa-miR-200a-3p	HMGB1	0[0,0]	0[0,0]	0[0,0]	0[0,0]	2[3,8]	2
hsa-miR-429	HMGB1	0[0,0]	0[0,0]	0[0,0]	1[1,5]	2[7,128]	2
hsa-miR-34a-5p	HMGB1	0[0,0]	0[0,0]	0[0,0]	0[0,0]	1[1,9]	2
hsa-miR-186-5p	HMGB1	0[0,0]	3[8,203]	0[0,0]	0[0,0]	0[0,0]	2
hsa-miR-181a-5p	HMGB1	0[0,0]	2[5,67]	0[0,0]	0[0,0]	0[0,0]	1
hsa-miR-202-3p	HMGB1	0[0,0]	0[0,0]	1[1,7]	0[0,0]	1[1,7]	1

Supplementary Figure 2

Supplementary figure 2. Characterization of the stable constructed HCC cell lines and candidate binding miRNAs.

(a) Stable HMGB1/RICTOR interference HCCLM3 and PLC/PRF/5 cells, and HMGB1/RICTOR 3'UTR overexpressed QSG-7701 cells were constructed by lentivirus system. RNA expression levels of HMGB1 and RICTOR were depicted by the relative quantity calculation using CT value. ***$p<0.001$. (b) Relative gray value of Western Blot assays to determine HMGB1 and RICTOR protein level in indicated cells. (c) Candidate miRNAs binding to HMGB1 and RICTOR mRNA were screened using CLIP database (http://starbase.sysu.edu.cn/). (d) RICTOR mRNA expression levels were determined in KD-NC, KD-HMGB1, OE-NC and OE-HMGB1 HCC cell lines with or without transfection with scrambled antagomir (negative control), antagomir-200a, antagomir-200b or antagomir-429.

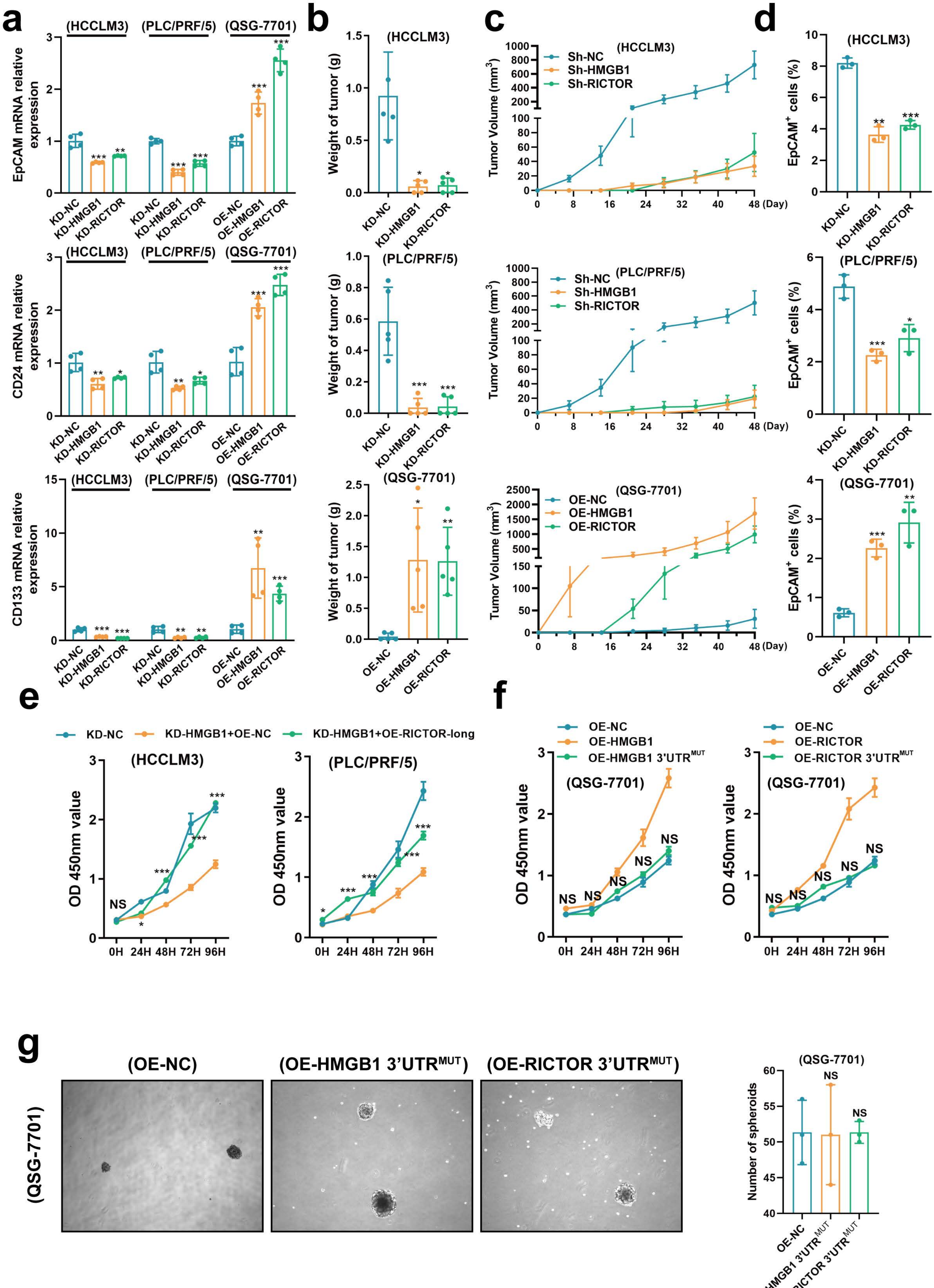

Supplementary Figure 3

Supplementary figure 3. HMGB1 epigenetically regulates stem-like properties of HCC cells.

(a) The CSCs markers in HCC cells were detected by real-time PCR. (b) The weight of the subcutaneous tumors was measured 48 days after the injection. (c) The growth curve of the subcutaneous tumors was depicted. (d) The percentage of $EPCAM^+$ HCC cells was determined by flow cytometry. (e) Malignant proliferation of HMGB1 interference HCC cells (KD-HMGB1 groups) transfected with full length RICTOR-overexpressed plasmid (noted as "OE-RICTOR-long") were determined using CCK-8 assay. (f-g) Cell proliferation (f) and spheroid formation (g) of liver cell lines transfected with wild-type ("OE-HMGB1" or "OE-RICTOR") or miR-200 family binding sites-mutated ("OE-HMGB1 3'UTR^{MUT}" or "OE-RICTOR 3'UTR^{MUT}") 3'UTR over-expression plasmids. NS: statistically non-significant when compared with "OE-NC" group.

a

Variable	Total cases (n=66)	PD-L1 expression (%)	
		Low	High
**BCLC stage**			
0+A	26	20 (77.9%)	6 (23.1%)
B+C	40	30 (75.0%)	10(25.0%)

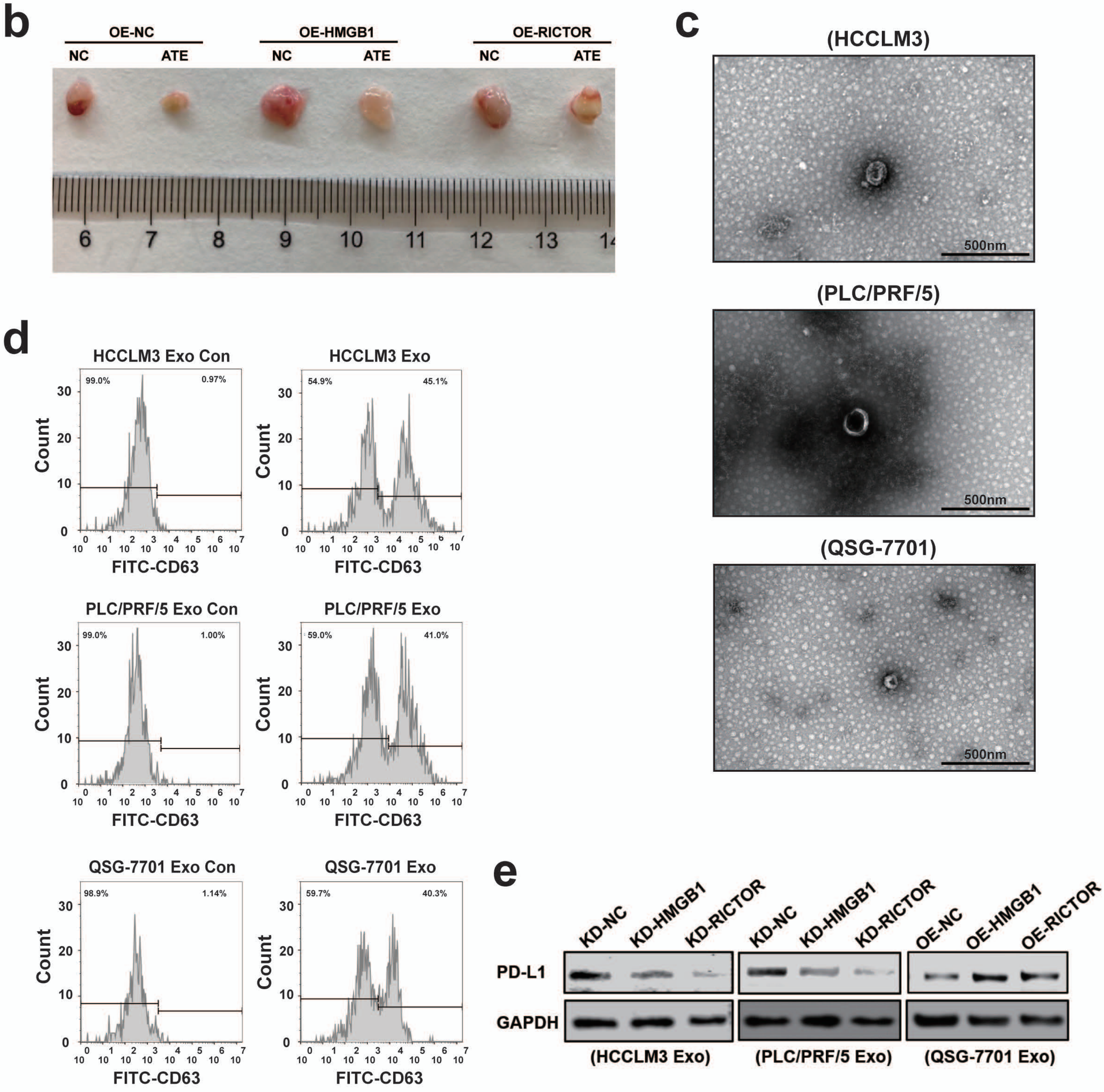

Supplementary Figure 4

Supplementary figure 4. HMGB1 and RICTOR mRNAs crosstalk regulates the response to anti-PD-L1 immunotherapy via upregulation of PD-L1^{+} exosomes in HCC.

(a) PD-L1 mRNA expression levels in different stages of HCC. (b) *In vivo* PMBC killing assay was performed in male NCG mice. (c) Representative electron microscopy images of exosomes secreted from parental cells. (d) Flow cytometry analysis of surface markers (CD63) on HCCLM3, PLC/PRF/5, and QSG-7701 cell-derived exosomes, respectively. (e) Exosomal protein level of PD-L1 determined by Western Blot.

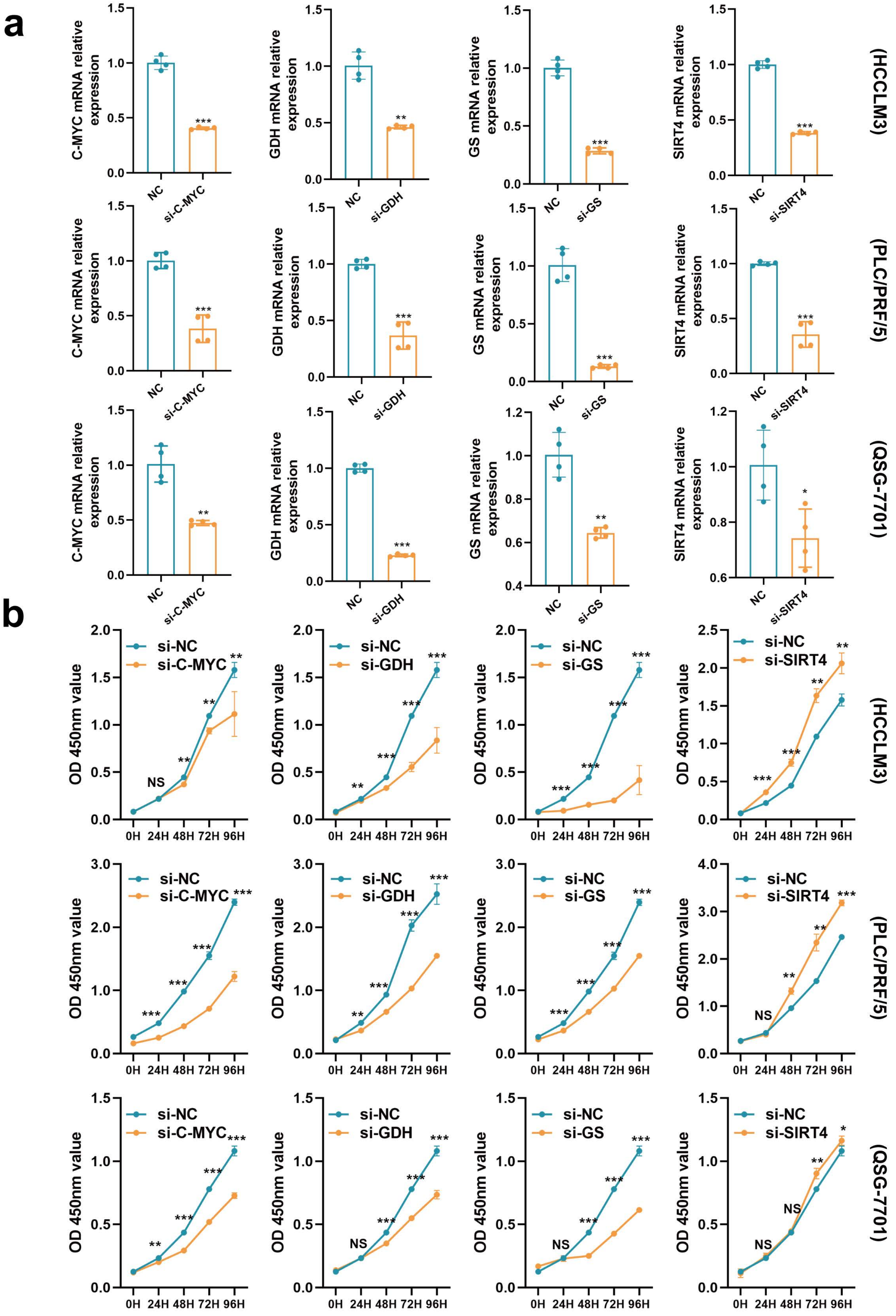

Supplementary Figure 5

Supplementary figure 5. The functions of C-MYC, GDH, GS, and SIRT4 in HCC. (a) Knockdown of C-MYC, GDH, GS, and SIRT4 in HCCLM3, PLC/PRF/5 and QSG-7701 cells using corresponding siRNAs. (b) CCK-8 assays were performed to determine malignant growth of HCC cells after C-MYC, GDH, GS, and SIRT4 knockdown, respectively.

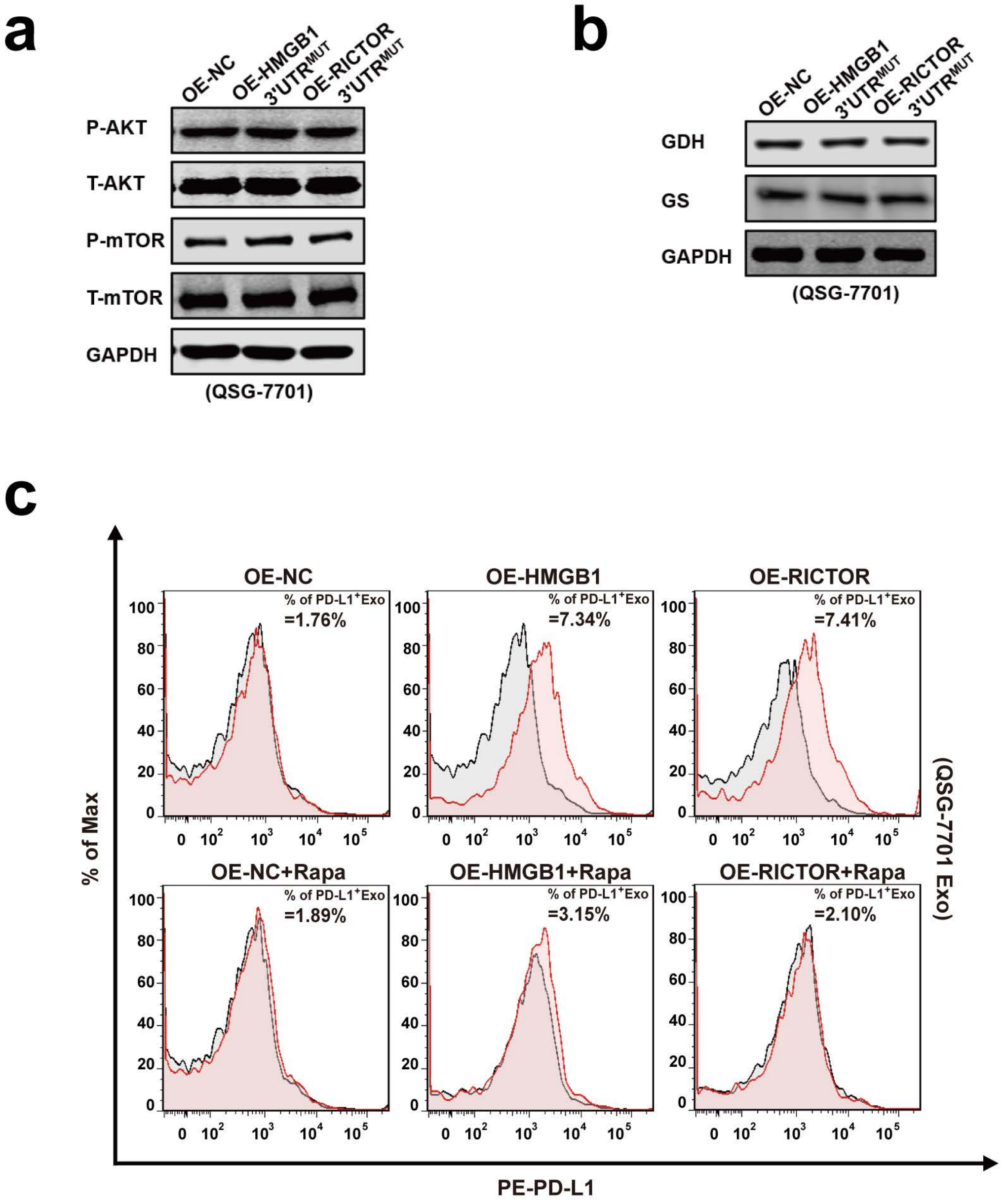

Supplementary Figure 6

Supplementary figure 6. HMGB1 crosstalk with RICTOR depends on miR-200 family binding sites and mTOR signaling to promote HCC progress.

(a-b) AKT-mTOR signaling pathway activation (a) and glutamine metabolism (b) after transfection with mutated-HMGB1/RICTOR 3'UTR overexpression plasmids. (c) Levels of PD-L1$^+$ exosomes were down-regulated after inhibition of mTORC1 by Rapamycin in OE-HMGB1/OE-RICTOR cell lines ("OE-HMGB1+Rapa"/"OE-RICTOR+Rapa"), when comparing to control group ("OE-HMGB1"/"OE-RICTOR"), respectively.

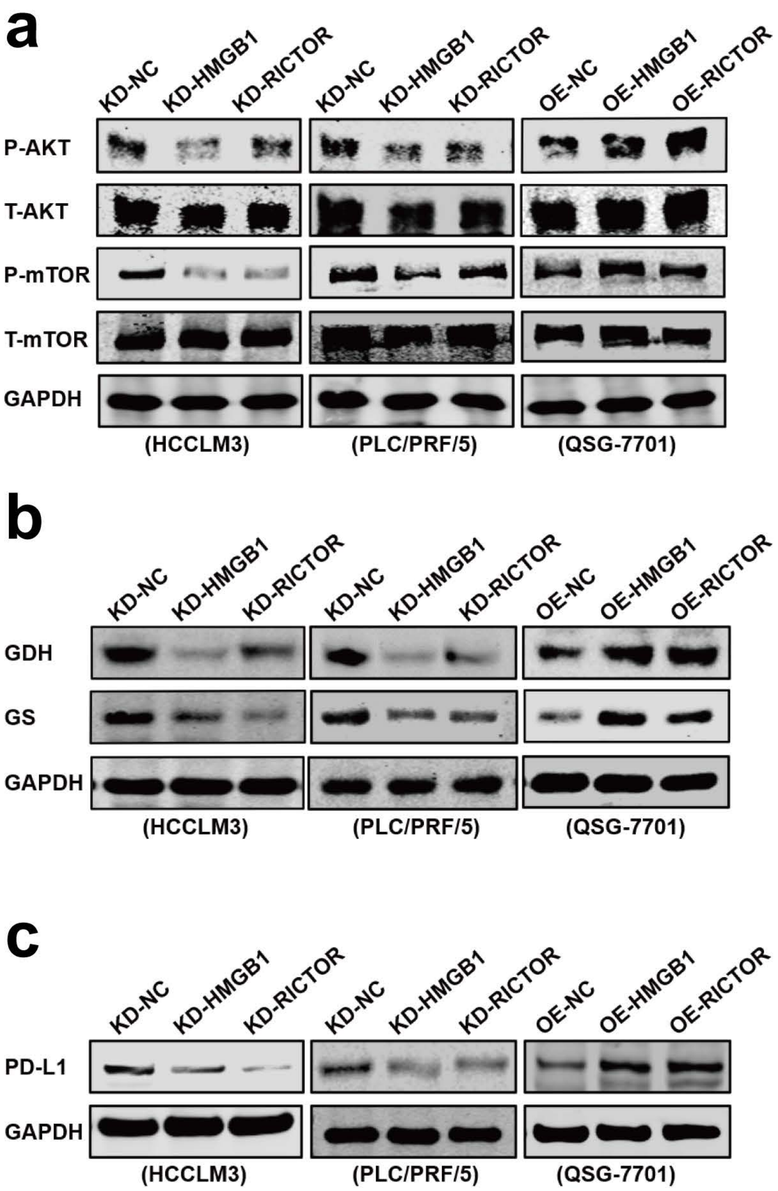

Supplementary Figure 7

Supplementary figure 7. Epigenetic crosstalk between HMGB1 and RICTOR stimulates glutamine metabolism and promotes PD-L1 expression via mTOR signaling in vivo.

(a-c) Phosphorylation of AKT and mTOR signaling (a), expressions of GS and GDH (b), and PD-L1 (c) in subcutaneously implanted tumors of HMGB1 and RICTOR-manipulated cell lines were determined.
